# Supplementary material for: The male bias of a generically-intended masculine pronoun: Evidence from eye-tracking and sentence evaluation
Source: PLoS One. 2021 Apr 1;16(4):e0249309. doi: 10.1371/journal.pone.0249309 (PMC8016286; doi:10.1371/journal.pone.0249309)
Supplement: S1 Table — (PDF) [file pone.0249309.s001.pdf]

S1 Table. Results of rating study for stereotypical activities. Ratings range from 1 corresponding to female to 7 corresponding to male.

Activity	M	SD	Literal translat. *noun*	Literal translat. *verb*	Translation
meidenavond plannen	1.196	0.483	girls' night	planning	planning a girls' night out
beha rechtdoen	1.196	0.585	bra	straightening	adjusting one's bra
bikini aantrekken	1.214	0.594	bikini	putting on	putting on a bikini
panty aandoen	1.286	0.530	tights	putting on	putting on tights
mascara opdoen	1.321	0.636	mascara	putting on	putting on mascara
trouwjurken bekijken	1.339	0.668	wedding dresses	viewing	looking at wedding dresses
zomerjurken inpakken	1.339	0.668	summer dresses	packing	packing sundresses
wimpers verven	1.339	0.611	eyelashes	painting	dyeing one's eyelashes
make-up bijwerken	1.339	0.640	make up	retouching	touching up one's make up
lippenstift bijwerken	1.411	0.682	lipstick	retouching	fixing one's lipstick
make-up opdoen	1.411	0.654	make up	putting on	putting on make up
tampons kopen	1.429	0.684	tampons	buying	buying tampons
nagels lakken	1.429	0.710	nails	painting	painting one's nails
krulspelden indoen	1.482	0.738	curlers	putting in	putting in hair rollers
lingerie wassen	1.571	0.735	lingerie	washing	washing lingerie
korset kopen	1.571	0.684	corset	buying	buying a corset
lippen verven	1.571	0.710	lips	painting	painting lips
buikdansles volgen	1.625	0.799	belly dance class	following	taking belly dance classes
benen scheren	1.625	0.728	legs	shaving	shaving one's legs
handtas inpakken	1.643	0.819	handbag	packing	packing one's handbag
haar vlechten	1.643	0.672	hair	braiding	braiding one's hair
breipatroon uitzoeken	1.679	0.741	knitting patterns	picking out	picking out knitting patterns
haar stijlen	1.696	0.761	hair	styling	straightening one's hair
modeblog updaten	1.714	0.847	fashion blog	updating	updating a fashion blog
breiwerk afmaken	1.714	0.731	knitwork	finishing	finishing the knitwork
Barbie aankleden	1.732	0.863	Barbie doll	dressing	dressing a Barbie doll
gezichtsmasker aanbrengen	1.768	0.809	face mask	applying	applying a face mask
wenkbrauwen epileren	1.768	0.853	eyebrows	plucking	plucking one's eyebrows
legging inpakken	1.768	0.809	tights	packing	packing a legging
sjaal breien	1.768	0.786	scarf	knitting	knitting a scarf
modetijdschrift lezen	1.821	0.789	fashion magazine	reading	reading a fashion magazine
nagels vijlen	1.875	0.854	nails	filing	filing one's nails
tas naaien	1.875	0.854	bag	sewing	sewing a bag
oorbellen indoen	1.911	0.900	earrings	putting in	putting on earrings
balletschoenen aantrekken	1.929	0.783	ballet shoes	putting on	putting on ballet shoes
naam borduren	1.929	0.828	name	embroiding	needlepointing a name
sierkussens kopen	1.946	0.862	decorative cushions	buying	buying decorative cushions
huishoudbeurs bezoeken	1.964	0.934	Huishoudbeurs	visiting	visiting the Huishoudbeurs (an annual Dutch fair for household products)

modeshow bekijken	1.964	0.808	fashion show	viewing	watching a fashion show
sieraden opbergen	1.982	0.904	jewelry	putting away	putting away jewelry
haarverf kopen	2.018	0.924	hair dye	buying	buying hair dye
bloemenkransen maken	2.036	0.830	flower wreaths	making	making flower wreaths
liefdesverhalen lezen	2.054	0.840	love stories	reading	reading love stories
yogabroek aandoen	2.071	0.931	yoga pants	putting on	putting on yoga pants
roddelblad lezen	2.089	0.745	gossip magazine	reading	reading a gossip magazine
hakschoenen poetsen	2.089	1.164	high heels	cleaning	cleaning high heels
naaimachine klaarzetten	2.107	0.888	sewing machine	setting up	setting up a sewing machine
pirouettes oefenen	2.107	0.779	pirouettes	practicing	practicing pirouettes
haar verven	2.125	0.916	hair	painting	dyeing one's hair
cupcakes versieren	2.161	0.869	cupcakes	decorating	decorating cupcakes
oksels scheren	2.161	0.910	armpits	shaving	shaving one's armpits
haar borstelen	2.161	0.910	hair	brushing	brushing one's hair
vlinders tekenen	2.179	0.811	butterflies	drawing	drawing butterflies
liefdesfilm kijken	2.232	0.809	love movie	watching	watching a romantic movie
bontjas aandoen	2.250	0.919	fur coat	putting on	putting on a fur coat
dagcrème aanbrengen	2.268	1.000	day cream	applying	applying day cream
haar föhnen	2.268	0.981	hair	blow-drying	blow-drying one's hair
theeblaadjes lezen	2.304	0.933	tea leaves	reading	reading tea leaves
dagboek bijhouden	2.304	0.872	diary	keeping up	keeping a diary
paard borstelen	2.321	0.855	horse	brushing	grooming a horse
yogaoefeningen doen	2.357	0.883	yoga exercises	doing	doing yoga exercises
vriendje bellen	2.375	1.214	boyfriend	calling	calling one's boyfriend
horoscoop lezen	2.375	0.843	horoscope	reading	reading the horoscope
roddels vertellen	2.411	1.058	gossip	telling	gossiping
lampionkettingen knutselen	2.429	0.931	lantern chains	crafting	crafting lantern chains
calorieën opschrijven	2.446	0.971	calories	writing down	writing down calories
paard opzadelen	2.446	0.933	horse	saddling	saddling horse
taart glaceren	2.446	0.913	cake	icing	icing a cake
danswedstrijd volgen	2.464	0.914	dance competition	following	following dance competition
boeket samenstellen	2.464	0.852	bouquet	putting together	putting together a bouquet
porselein beschilderen	2.464	0.953	china	painting	painting china
soap kijken	2.482	0.894	soap opera	watching	watching a soap opera
baby voeden	2.482	1.044	baby	feeding	feeding a baby
sandaaltjes kopen	2.500	1.265	sandals	buying	buying sandals
hart uitstorten	2.500	0.915	heart	pouring out	pouring one's heart out
accessoires uitkiezen	2.518	0.934	accessories	picking out	picking out accessories
koekjes bakken	2.536	0.894	cookies	baking	baking cookies
kapsels bekijken	2.554	1.043	haircuts	viewing	looking at haircuts
dromen opschrijven	2.554	1.043	dreams	writing down	writing down one's dreams
kaarsen aansteken	2.589	0.910	candles	lighting	lighting candles
wierook aansteken	2.607	1.139	incense	lighting	lighting incense
relatieproblemen bespreken	2.625	0.926	relationship problems	discussing	discussing relationship problems
baby bewonderen	2.625	1.071	baby	admiring	admiring a baby

paard bestijgen	2.643	0.883	horse	mounting	mounting a horse
musicallessen volgen	2.696	0.952	musical lessons	following	taking musical lessons
babytaal spreken	2.696	1.094	baby language	speaking	speaking baby language
harp bespelen	2.696	0.851	harp	playing	playing the harp
dieet volgen	2.696	0.851	diet	following	following diet
verjaardagskaart tekenen	2.714	1.039	birthday card	singing	singing birthday card
handlijnen lezen	2.732	1.000	hand lines	reading	reading hand lines
kooktijdschrift doorbladeren	2.750	0.995	cooking magazine	browsing	browsing a cooking magazine
bonbons eten	2.786	0.847	chocolates	eating	eating chocolates
luiers verschonen	2.786	0.803	diapers	changing	changing diapers
huwelijksplannen maken	2.804	0.923	wedding plans	making	making wedding plans
heupen trainen	2.804	1.135	hips	training	training hips
toonladders zingen	2.821	0.993	scales	singing	singing scales
huis schoonmaken	2.821	0.876	house	cleaning	cleaning the house
thee drinken	2.821	0.855	tea	drinking	drinking tea
fotoboeken bekijken	2.839	0.968	photo books	viewing	looking at photo books
chocola eten	2.839	0.848	chocolate	eating	eating chocolate
outfit samenstellen	2.857	0.773	outfit	putting together	putting together an outfit
schaatskunstjes perfectioneren	2.875	0.992	ice skating tricks	perfecting	perfecting ice skating tricks
tarotkaarten leggen	2.875	0.935	tarot cards	laying	laying tarot cards
was doen	2.875	0.955	laundry	doing	doing laundry
vloer dweilen	2.875	0.955	floor	mopping	mopping the floor
cadeaus inpakken	2.929	0.970	presents	wrapping	wrapping presents
parfum opdoen	2.946	0.999	perfume	putting on	putting on perfume
bed opmaken	2.946	0.818	bed	making up	making the bed
kleurplaat inkleuren	2.964	0.894	coloring page	coloring (in)	coloring a picture
chocoladereep eten	2.964	0.785	chocolate bar	eating	eating a candy bar
ramen schoonmaken	3.000	1.206	windows	cleaning	cleaning windows
stem opwarmen	3.000	0.972	voice	warming up	warming up one's voice
prosecco drinken	3.018	1.104	prosecco	drinking	drinking prosecco
kerstboom versieren	3.054	0.999	Christmas tree	decorating	decorating Christmas tree
liedjes zingen	3.054	0.840	songs	singing	singing songs
spaarkaart inruilen	3.089	1.014	savings card	exchanging	exchanging a savings card
bubbelbad nemen	3.089	0.793	bubble bath	taking	taking a bubble bath
karaoke zingen	3.089	0.959	karaoke	singing	singing karaoke
avondeten klaarmaken	3.107	0.888	dinner	preparing	preparing dinner
boekenbonnen inleveren	3.125	0.974	book vouchers	handing in	redeem book vouchers
kinderen ophalen	3.125	0.955	children	picking up	picking up the children
boodschappen doen	3.143	0.980	groceries	doing	getting groceries
gedichten lezen	3.143	1.034	poems	reading	reading poetry
koffiedik kijken	3.161	1.125	coffee grounds	watching	reading the tea leaves
tanden bleken	3.161	0.987	teeth	bleaching	whitening one's teeth
foto's maken	3.179	0.974	pictures	making	taking pictures
kinderen wegbrengen	3.196	0.961	children	taking away	dropping off the children
schoolfeest voorbereiden	3.214	0.909	school party	preparing	preparing school party

surprise maken	3.232	0.809	surprise	making	making a surprise gift
ouderschapsverlof regelen	3.268	1.120	parental leave	arranging	arranging parental leave
pralines eten	3.286	0.780	chocolates	eating	eating chocolates
kind voorlezen	3.286	0.929	child	reading to	reading to a child
spiegelbeeld bestuderen	3.304	0.893	reflection	studying	studying one's reflection
haar wassen	3.321	0.917	hair	washing	washing one's hair
brief schrijven	3.357	0.923	letter	writing	writing a letter
geld uitgeven	3.357	1.017	money	spending	spending money
kat aaien	3.357	0.819	cat	petting	petting a cat
levensverhaal vertellen	3.375	1.088	life story	telling	telling one's life story
passagiers uitzwaaien	3.375	0.885	passengers	wave goodbye	waving goodbye to passengers
ouders bezoeken	3.375	0.843	parents	visiting	visiting one's parents
boek lezen	3.393	0.908	book	reading	reading a book
volleybaloefeningen doen	3.411	0.910	volleyball exercises	doing	doing volleyball exercises
verlanglijst maken	3.464	0.713	wish list	making	making wish list
schilderij maken	3.464	0.762	painting	making	making painting
ramen opendoen	3.518	0.972	windows	opening	opening the windows
bureau opruimen	3.518	0.934	desk	tidying up	tidying a desk
maan bekijken	3.536	1.095	moon	viewing	watching the moon
handen wassen	3.554	0.685	hands	washing	washing one's hands
tentoonstelling bekijken	3.571	0.783	exhibition	viewing	checking out an exhibition
woordenschat oefenen	3.571	0.783	vocabulary	practicing	practicing vocabulary
popliedjes luisteren	3.571	0.684	pop songs	listening	listening to pop songs
vakantie plannen	3.571	1.093	vacation	planning	planning a vacation
koffers pakken	3.589	0.949	suitcases	taking	taking suitcases
taart eten	3.589	0.804	cake	eating	having cake
Facebook checken	3.607	0.679	Facebook	checking	checking Facebook
telefoon checken	3.607	0.731	phone	checking	checking one's phone
spullen pakken	3.607	0.755	stuff	taking	taking stuff
bezoek begroeten	3.625	0.728	visitor/visitors	greet	greeting a visitor
hockeyschoenen aandoen	3.625	0.843	hockey shoes	putting on	putting on hockey shoes
reisinformatie bestuderen	3.679	1.011	travel information	studying	studying travel information
brood smeren	3.679	0.917	bread	buttering	preparing a sandwich
presentatie voorbereiden	3.696	0.658	presentation	preparing	preparing a presentation
kater zoeken	3.696	0.913	tomcat	searching	looking for one's tomcat
opdracht maken	3.714	0.680	assignment	making	making assignment
paraplu openklappen	3.714	0.868	umbrella	folding open	opening an umbrella
Sinterklaas vieren	3.732	0.674	Sinterklaas	celebrating	celebrating Sinterklaas
ruzie maken	3.750	0.815	argument	making	having an argument
uitzicht bewonderen	3.750	0.667	view	admiring	admiring the view
lenzen indoen	3.768	0.713	contact lenses	putting in	putting in contact lenses
kaartje printen	3.768	0.853	ticket	printing	printing a ticket
appelflap eten	3.786	0.624	apple turnover	eating	eating an apple turnover
gordel vastmaken	3.804	0.483	seat belt	attaching	fastening the seat belt

puinzooi opruimen	3.804	1.482	mess	cleaning up	cleaning up a mess
schoenen aandoen	3.839	0.682	shoes	putting on	putting on shoes
werkstuk schrijven	3.857	0.520	paper	writing	writing a paper
telefoon opladen	3.875	0.541	phone	charging	charging one's phone
lunch eten	3.875	0.541	lunch	eating	having lunch
jas aandoen	3.875	0.541	coat	putting on	putting on a coat
boardingpass printen	3.893	1.107	boarding pass	printing	printing a boarding pass
rooster samenstellen	3.893	0.928	schedule	putting together	putting together a schedule
bagage inchecken	3.893	0.947	luggage	checking in	checking in luggage
cijfers bekijken	3.893	0.731	grades	viewing	checking one's grades
tanden poetsen	3.893	0.366	teeth	cleaning	brushing one's teeth
tentamen maken	3.893	0.493	exam	making	taking an exam
post lezen	3.893	0.824	mail	reading	reading mail
OV-kaart opladen	3.911	0.668	public transport card	charging	topping up the balance on a public transport card
zonnebril opzetten	3.911	0.745	sunglasses	putting on	putting on sunglasses
antwoord opschrijven	3.911	0.478	answer	writing down	writing down the answer
rijlessen inplannen	3.929	0.806	driving lessons	scheduling	scheduling driving lessons
cocktails mixen	3.929	1.126	cocktails	mixing	mixing cocktails
zitplaats zoeken	3.946	0.699	seat	searching	looking for a seat
ballon opblazen	3.964	0.873	balloon	blowing up	blowing up a balloon
sherry drinken	3.964	1.361	sherry	drinking	drinking sherry
kerstpakket afhalen	3.982	0.904	Christmas package	picking up	picking up a Christmas package
muziek luisteren	3.982	0.522	music	listening	listening to music
paspoort zoeken	4.000	0.972	passport	searching	looking for one's passport
tennispartner zoeken	4.018	0.863	tennis partner	searching	looking for a tennis partner
stembiljet invullen	4.018	0.774	voting ballot	filling out	marking a ballot
wachtwoord wijzigen	4.018	0.842	password	changing	changing one's password
koelkast ontdooien	4.018	1.053	fridge	defrosting	defrosting the fridge
neus snuiten	4.018	0.447	nose	blowing	blowing one's nose
treinkaartje kopen	4.036	0.687	train ticket	buying	buying a train ticket
CV opstellen	4.036	0.914	resume	drafting	drafting a resume
shotjes doen	4.036	0.762	shots	doing	drinking shots
pauze nemen	4.036	0.631	pause	taking	taking a break
radio luisteren	4.054	0.553	radio	listening	listening to the radio
documentaire kijken	4.071	0.871	documentary	watching	watching a documentary
frustratie uiten	4.071	1.042	frustration	expressing	expressing frustration
veters strikken	4.071	0.499	shoelaces	tying	tying one's shoelaces
patiënten behandelen	4.071	0.759	patients	treating	treating (one's) patients
weerbericht kijken	4.107	0.755	weather forecast	watching	watching the weather forecast
carnaval vieren	4.107	0.412	carnival	celebrating	celebrating carnival
tv kijken	4.107	0.779	TV	watching	watching TV
fietsroute opzoeken	4.143	1.119	cycle route	looking up	looking up cycle route
oplossing zoeken	4.143	0.923	solution	searching	finding a solution
skipasje scannen	4.161	0.733	ski pass	scanning	scanning a ski pass

ski's aandoen	4.161	0.532	skis	putting on	putting on skis
onderzoek doen	4.179	0.765	research	doing	doing research
koffie opdrinken	4.179	0.741	coffee	drinking up	finishing the coffee
laptop opstarten	4.179	0.690	laptop	starting up	starting up a laptop
werkmails beantwoorden	4.196	0.699	work e-mails	answering	answering work e-mails
huur overmaken	4.214	1.057	rent	transferring	paying rent
baas bellen	4.214	0.624	boss	calling	calling the boss
regenbroek aantrekken	4.232	0.914	rain pants	putting on	putting on rain pants
vriendinnetje trakteren	4.250	1.871	girlfriend	treating	treating one's girlfriend (to something)
journaal kijken	4.250	0.694	journal	watching	watching the news
fiets stallen	4.250	0.769	bicycle	storing	storing a bike
hond uitlaten	4.304	0.761	dog	letting out	walking the dog
breakdancelessen volgen	4.321	1.146	breakdance lessons	following	following breakdance lessons
pakketten afhalen	4.357	1.135	packages	picking up	picking up packages
rekeningen betalen	4.357	1.135	bills	paying	paying the bills
buikspieren trainen	4.393	1.123	abdominal muscles	training	training one's abs
bowlingschoenen aandoen	4.393	0.705	bowling shoes	putting on	putting on bowling shoes
tranen onderdrukken	4.393	1.423	tears	suppressing	holding back tears
data analyseren	4.429	0.759	data	analyzing	analyzing data
krant lezen	4.464	0.808	newspaper	reading	reading the newspaper
doktersjas aandoen	4.482	1.027	doctor's coat	putting on	putting on a doctor's coat
gitaar spelen	4.482	0.738	guitar	playing	playing the guitar
pokerface oefenen	4.536	0.972	poker face	practicing	practicing one's poker face
lawaai maken	4.536	1.008	noise	making	making noise
marathon lopen	4.554	0.829	marathon	walking	running a marathon
grapjes maken	4.607	0.755	jokes	making	telling jokes
liedje fluiten	4.607	0.888	song	whistling	whistling a song
postzegels organiseren	4.696	1.060	stamps	organizing	stamps
trainingsplan uitwerken	4.768	0.972	training plan	working out	developing a training plan
broek afritsen	4.768	1.279	pants	zipping off	zipping off one's pants
afval wegbrengen	4.786	1.187	garbage	taking away	disposing of garbage
parachute openen	4.857	0.943	parachute	opening	opening the parachute
uniform aandoen	4.857	0.980	uniform	putting on	putting on a uniform
gaspedaal indrukken	4.893	1.021	accelerator pedal	pushing in	pressing on the gas
vogels spotten	4.893	1.021	birds	spotting	bird watching
muren verven	4.929	0.970	walls	painting	painting walls
goocheltrucs oefenen	4.946	0.724	magic tricks	practicing	practicing magic tricks
klimschoenen aandoen	4.964	0.972	climbing shoes	putting on	putting on climbing shoes
woede afreageren	4.964	0.934	anger	abreacting	to vent one's anger
buit verstoppen	4.964	1.061	loot	hiding	hiding the loot
berg beklimmen	5.036	0.914	mountain	climbing	climbing a mountain
basgitaar stemmen	5.054	0.818	bass guitar	tuning	tuning a bass guitar
stripverhalen lezen	5.054	0.749	comics	reading	reading comics
inzet verhogen	5.054	0.840	bet	increasing	increasing the bet

veldbed opzetten	5.071	1.042	camp bed	setting up	setting up a camp bed
maten waarschuwen	5.071	1.219	pals	warning	warning one's pals
tent opzetten	5.071	0.931	tent	setting up	pitching a tent
werkbroek aandoen	5.089	0.978	overalls	putting on	putting on overalls
wiet roken	5.089	0.940	weed	smoking	smoking weed
fietsbanden oppompen	5.107	0.985	bicycle tires	inflating	inflating bicycle tires
spareribs eten	5.107	0.985	spare ribs	eating	eating spare ribs
surfplank waxen	5.125	1.028	surf board	waxing	waxing a surf board
drugs smokkelen	5.143	1.034	drugs	smuggling	smuggling drugs
golfbal slaan	5.161	0.781	golf ball	hitting	hitting a golf ball
zeilen hijsen	5.161	0.930	sails	hoisting	hoisting the sails
scheerapparaat reinigen	5.179	1.503	razor	cleaning	cleaning a razor
weddenschap afsluiten	5.179	0.897	bet	shutting down	placing a bet
keu krijten	5.179	1.208	cue	chalking	chalking a pool stick
metal luisteren	5.214	1.004	metal	listening	listening to metal music
club toejuichen	5.250	0.939	club	cheering on	cheering on a sports team
golfclubs poetsen	5.268	0.944	golf clubs	cleaning	cleaning golf clubs
vliegtuigjes vouwen	5.304	0.872	planes	folding	folding paper planes
auto wassen	5.304	1.008	car	washing	washing a car
bier drinken	5.321	0.956	beer	drinking	having a beer
superheldenfilm kijken	5.357	0.862	super hero movie	watching	watching a super hero movie
paintball spelen	5.357	0.903	paint ball	playing	playing paint ball
western kijken	5.357	0.923	western	watching	watching a western movie
geld vergokken	5.357	0.862	money	gambling away	gambling away money
blikje adten	5.357	0.923	can	chugging	chugging a can
bierglas leegdrinken	5.375	0.906	beer glass	drinking empty	finishing one's beer
legerkistjes poetsen	5.411	1.203	combat boots	cleaning	cleaning combat boots
ontvoering plannen	5.429	1.189	kidnapping	planning	planning a kidnapping
gras maaien	5.429	0.931	grass	mowing	mowing the lawn
krachtoefeningen doen	5.446	0.913	strength exercises	doing	doing strength exercises
computerspelletjes spelen	5.446	0.913	computer games	playing	playing computer games
stormschade opruimen	5.446	1.043	storm damage	cleaning up	cleaning up storm damages
sneeuw scheppen	5.446	0.952	snow	shoveling	shoveling snow
fietsband plakken	5.464	0.953	bicycle tire	sticking	patching a bicycle tire
aandelen verkopen	5.500	0.972	shares	selling	selling shares
mountainbike afstellen	5.518	0.972	mountain bike	adjusting	adjusting a mountain bike
voetbalschoenen aandoen	5.518	0.914	soccer shoes	putting on	putting on soccer shoes
pet opdoen	5.518	0.853	cap	putting on	putting on a cap
hengel uitwerpen	5.536	0.972	fishing rod	ejecting	casting out a fishing rod
bokshandschoenen aandoen	5.571	0.912	boxing gloves	putting on	putting on boxing gloves
vriendinnetje zoenen	5.589	1.262	girlfriend	kissing	kissing one's girlfriend
vuurwerk afsteken	5.589	0.869	fireworks	standing out	lighting fireworks
push-ups doen	5.589	0.949	push-ups	doing	doing push-ups
sportauto parkeren	5.607	0.966	sports car	parking	parking a sports car
racefiets opknappen	5.625	1.105	racing bike	fixing up	fixing up a racing bike

vogelhuisje timmeren	5.625	0.926	birdhouse	woodworking	building a birdhouse
Ferrari bewonderen	5.643	0.943	Ferrari	admiring	admiring a Ferrari
whisky drinken	5.643	0.903	whiskey	drinking	drinking whiskey
controller wegsmijten	5.661	1.083	controller	throwing away	throwing away a game controller
voetbalwedstrijd kijken	5.679	0.855	soccer game	watching	watching a soccer game
visnet uitgooien	5.679	0.993	fishing net	throwing out	throwing out a fishing net
skateboardtrucs doen	5.696	0.913	skateboard tricks	doing	doing skateboard tricks
meubels monteren	5.696	0.971	furniture	assembling	assembling furniture
geweer laden	5.696	0.933	gun	loading	loading a gun
motorvakantie plannen	5.714	0.909	motorcycle vacation	planning	planning a motorcycle vacation
boomhut bouwen	5.714	0.847	treehouse	building	building a treehouse
bankoverval voorbereiden	5.732	1.036	bank robbery	preparing	preparing a bank robbery
bokszak ophangen	5.750	0.958	boxing bag	hanging up	hanging up boxing bag
biljart spelen	5.750	0.919	billiards	playing	playing billiards
barbecue aansteken	5.768	0.874	barbecue	lighting	lighting a barbecue
gereedschap klaarleggen	5.786	0.967	tools	laying out	laying out tools
bouwhelm vastmaken	5.804	0.840	construction helmet	attaching	fastening a construction helmet
zwembroek aandoen	5.804	1.119	swimming pants	putting on	putting on a swimsuit
oliepeil controleren	5.804	0.942	oil level	checking	checking the oil level
biceps trainen	5.804	0.903	biceps	training	training one's biceps
lasbril opdoen	5.821	0.855	welding goggles	putting on	putting on welding goggles
modelvliegtuig starten	5.821	1.011	model airplane	starting	starting a model airplane
bokswedstrijd kijken	5.821	1.011	boxing match	watching	watching a boxing match
gewichten heffen	5.821	0.897	weights	lifting	lifting weights
overhemd aandoen	5.839	0.890	shirt	putting on	putting on a shirt
planken zagen	5.857	0.862	planks	sawing	cutting boards
voetbaltrucs oefenen	5.893	0.824	soccer tricks	practicing	practicing soccer tricks
autobanden verwisselen	5.893	0.802	car tires	swap	changing car tires
schoorsteen vegen	5.893	0.928	chimney	sweeping	sweeping a chimney
pistool reinigen	5.893	0.985	pistol	cleaning	cleaning a gun
voetbalplaatjes uitwisselen	5.911	0.859	soccer images	exchanging	trading soccer cards
hout zagen	5.911	0.959	wood	sawing	sawing wood
das strikken	5.911	1.164	tie	tying	tying a tie
modeltreinen besturen	5.929	0.931	model trains	driving	playing with model trains
bekabeling controleren	5.929	0.912	wiring	checking	check the wiring
borstspieren trainen	5.946	0.883	chest muscles	training	training one's chest muscles
tafel timmeren	5.946	0.903	table	woodworking	building a table
haargel indoen	5.964	0.894	hair gel	putting in	putting in hair gel
muur afbreken	5.982	0.904	wall	tearing down	tearing down a wall
bier brouwen	5.982	0.924	beer	brewing	brewing beer
scooter opvoeren	6.000	0.874	scooter	tuning	tuning a scooter
sigaar roken	6.000	0.934	cigar	smoking	smoking a cigar
pak aandoen	6.000	0.831	suit	putting on	putting on a suit
bouwklus afronden	6.018	0.798	building job	finishing	finishing a building job

bierbuik wegwerken	6.018	1.018	beer belly	getting rid of	getting rid of one's beer belly
hout hakken	6.018	0.820	wood	chopping	chopping wood
Formule 1 kijken	6.036	0.873	Formula 1	watching	watching Formula 1
auto repareren	6.054	0.903	car	repairing	repairing a car
tanks bewonderen	6.071	0.783	tanks	admiring	admiring army tanks
tractor repareren	6.089	0.978	tractor	repairing	repairing a tractor
sloopwerk doen	6.125	0.896	demolition work	doing	doing demolition work
pijp roken	6.161	0.949	pipe	smoking	smoking a pipe
dak repareren	6.179	0.855	roof	repairing	repairing the roof
beton gieten	6.214	0.868	concrete	pouring	pouring concrete
borst scheren	6.304	0.851	chest	shaving	shaving one's chest
snor scheren	6.393	0.846	moustache	shaving	shaving one's moustache
Playboy lezen	6.411	0.733	Playboy	reading	reading the Playboy
mannenavond plannen	6.804	0.553	men's night	planning	planning a guys' night out
