# Supplementary material for: The male bias of a generically-intended masculine pronoun: Evidence from eye-tracking and sentence evaluation
Source: PLoS One. 2021 Apr 1;16(4):e0249309. doi: 10.1371/journal.pone.0249309 (PMC8016286; doi:10.1371/journal.pone.0249309)
Supplement: S2 Table — (PDF) [file pone.0249309.s002.pdf]

S2 Table. Activities used for experimental stimuli with their mean rating and standard deviation on a 7-point scale, with 1 representing female and 7 representing male.

**Female stereotype**	***M***	***SD***	**Male stereotype**	***M***	**SD**	**Neutral stereotype**	***M***	**SD**
breiwerk afmaken 'finishing the knitwork'	1.71	0.73	*dak repareren* 'repairing the roof'	6.18	0.86	*gordel vastmaken* 'fastening the seat belt'	3.80	0.48
wenkbrauwen epileren 'plucking one's eyebrows'	1.77	0.85	*pijp roken* 'smoking a pipe'	6.16	0.95	*schoenen aandoen* 'putting on shoes'	3.84	0.68
oorbellen indoen 'putting on earrings'	1.91	0.90	*auto repareren* 'repairing a car'	6.05	0.90	*jas aandoen* 'putting on a coat'	3.88	0.54
balletschoenen aantrekken 'putting on ballet shoes'	1.93	0.78	*bouwklus afronden* 'finishing a building job'	6.02	0.80	*lunch eten* 'having lunch'	3.88	0.54
naam borduren 'needlepointing a name'	1.93	0.83	*pak aandoen* 'putting on a suit'	6.00	0.83	*telefoon opladen* 'charging one's phone'	3.88	0.54
sieraden opbergen 'putting away jewelry'	1.98	0.90	*sigaar roken* 'smoking a cigar'	6.00	0.93	*bagage inchecken* 'checking in luggage'	3.89	0.95
roddelblad lezen 'reading a gossip magazine'	2.09	0.75	*borstspieren trainen* 'training one's chest muscles'	5.95	0.88	*cijfers bekijken* 'checking one's grades'	3.89	0.73
naaimachine klaarzetten 'setting up a sewing machine'	2.11	0.89	*autobanden verwisselen* 'changing car tires'	5.89	0.80	*rooster samenstellen* 'putting together a schedule'	3.89	0.93
pirouettes oefenen 'practicing pirouettes'	2.11	0.78	*pistool reinigen* 'cleaning a gun'	5.89	0.98	*tanden poetsen* 'brushing one's teeth'	3.89	0.37
haar verven 'dyeing one's hair'	2.13	0.92	*voetbaltrucs oefenen* 'practicing soccer tricks'	5.89	0.82	*tentamen maken* 'taking an exam'	3.89	0.49
cupcakes versieren 'decorating cupcakes'	2.16	0.87	*bouwhelm vastmaken* 'fastening a construction helmet'	5.80	0.84	*antwoord opschrijven* 'writing down the answer'	3.91	0.48
oksels scheren 'shaving one's armpits'	2.16	0.91	*oliepeil controleren* 'checking the oil level'	5.80	0.94	*rijlessen inplannen* 'scheduling driving lessons'	3.93	0.81
dagcrème aanbrengen 'applying day cream'	2.27	1.00	*gereedschap klaarleggen* 'laying out tools'	5.79	0.97	*paspoort zoeken* 'looking for one's passport'	4.00	0.97
dagboek bijhouden 'keeping a diary'	2.30	0.87	*motorvakantie plannen* 'planning a motorcycle vacation'	5.71	0.91	*neus snuiten* 'blowing one's nose'	4.02	0.45
paard borstelen 'grooming a horse'	2.32	0.86	*geweer laden* 'loading a gun'	5.70	0.93	*stembiljet invullen* 'marking a ballot'	4.02	0.77
yogaoefeningen doen 'doing yoga exercises'	2.36	0.88	*sportauto parkeren* 'parking a sports car'	5.61	0.97	*wachtwoord wijzigen* 'changing one's password'	4.02	0.84
horoscoop lezen 'reading the horoscope'	2.38	0.84	*bokshandschoenen aandoen* 'putting on boxing gloves'	5.57	0.91	*CV opstellen* 'drafting a resume'	4.04	0.91
calorieën opschrijven 'writing down calories'	2.45	0.97	*hengel uitwerpen* 'casting out a fishing rod'	5.54	0.97	*patiënten behandelen* 'treating patients'	4.07	0.76
hart uitstorten 'pouring one's heart out'	2.50	0.91	*mountainbike afstellen* 'adjusting a mountain bike'	5.52	0.97	*veters strikken* 'tying one's shoelaces'	4.07	0.50

kaarsen aansteken 'lighting candles'	2.59	0.91	*voetbalschoenen aandoen* 'putting on soccer shoes'	5.52	0.91	*ski's aandoen* 'putting on skis'	4.16	0.53
relatieproblemen bespreken 'discussing relationship problems'	2.63	0.93	*krachtoefeningen doen* 'doing strength exercises'	5.45	0.91	*koffie opdrinken* 'finishing the coffee'	4.18	0.74
thee drinken 'drinking tea'	2.82	0.86	*blikje adten* 'chugging a can'	5.36	0.92	*laptop opstarten* starting up a laptop'	4.18	0.69
outfit samenstellen 'putting together an outfit'	2.86	0.77	*tent opzetten* 'pitching a tent'	5.07	0.93	*werkmails beantwoorden* 'answering work e-mails'	4.20	0.70
was doen 'doing laundry'	2.88	0.95	*inzet verhogen* 'increasing the bet'	5.05	0.84	*fiets stallen* 'storing a bike'	4.25	0.77
TOTAL	2.26	0.33	*TOTAL*	5.73	0.31	*TOTAL*	3.99	0.13
