# Supplementary material for: The male bias of a generically-intended masculine pronoun: Evidence from eye-tracking and sentence evaluation
Source: PLoS One. 2021 Apr 1;16(4):e0249309. doi: 10.1371/journal.pone.0249309 (PMC8016286; doi:10.1371/journal.pone.0249309)
Supplement: S1 Stimuli — (PDF) [file pone.0249309.s003.pdf]

S1 Stimuli. Stimuli for Experiment 1. All stimuli are provided in the experimental condition with a female continuation and the quantifier *enkele* ‘some’. Stimuli for Experiment 2 were the same as below, but shortened by replacing everything starting from “, maar” with a period.

**Nr.**	**Stereotype**	**Stimulus**
1	neutral	Iedereen was zijn gordel aan het vastmaken, waaronder enkele vrouwen die al een tijdje in het vliegtuig zaten te wachten, maar nu eindelijk konden vertrekken.
2	neutral	Iedereen was zijn schoenen aan het aandoen, waaronder enkele vrouwen die al bijna klaar waren om de deur uit te gaan, maar een beetje aan het treuzelen waren.
3	neutral	Iedereen was zijn jas aan het aandoen, waaronder enkele vrouwen die al een tijdje hadden uitgekeken naar een wandeling in de zon, maar eerst nog hadden vergaderd.
4	neutral	Iedereen was zijn lunch aan het eten, waaronder enkele vrouwen die al eerder honger hadden gekregen, maar nog een belangrijke afspraak gehad hadden.
5	neutral	Iedereen was zijn telefoon aan het opladen, waaronder enkele vrouwen die al een hele dag met een lege batterij hadden rondgelopen, maar niet eerder een stopcontact waren tegengekomen op het festivalterrein.
6	neutral	Iedereen was zijn bagage aan het inchecken, waaronder enkele vrouwen die al over dertig minuten bij de gate moesten zijn, maar nog steeds bij de incheckbalie stonden.
7	neutral	Iedereen was zijn cijfers aan het bekijken, waaronder enkele vrouwen die al gelijk na het mondeling wisten dat ze het vak gehaald hadden, maar toch heel benieuwd waren.
8	neutral	Iedereen was zijn rooster aan het samenstellen, waaronder enkele vrouwen die al snel de eerste periode hadden volgepland, maar er voor de tweede periode nog niet uit waren.
9	neutral	Iedereen was zijn tanden aan het poetsen, waaronder enkele vrouwen die al een jaar niet meer bij de tandarts waren geweest, maar er nu aan moesten geloven.
10	neutral	Iedereen was zijn tentamen aan het maken, waaronder enkele vrouwen die al in het vierde jaar zaten, maar deze toets nog niet gehaald hadden.
11	neutral	Iedereen was zijn antwoord aan het opschrijven, waaronder enkele vrouwen die al vaker de pubquiz hadden gewonnen, maar deze week minder goed presteerden.
12	neutral	Iedereen was zijn rijlessen aan het inplannen, waaronder enkele vrouwen die al een keer examen hadden gedaan, maar toen gezakt waren.
13	neutral	Iedereen was zijn paspoort aan het zoeken, waaronder enkele vrouwen die al bijna moesten boarden, maar nog steeds niet alle papieren hadden gevonden.
14	neutral	Iedereen was zijn neus aan het snuiten, waaronder enkele vrouwen die al twee weken verkouden waren, maar nog niet naar de dokter waren geweest.

15	neutral	Iedereen was zijn stembiljet aan het invullen, waaronder enkele vrouwen die al twee keer de stemwijzer hadden gedaan, maar nog steeds stonden te twijfelen.
16	neutral	Iedereen was zijn wachtwoord aan het wijzigen, waaronder enkele vrouwen die al twee keer een herinnering per e-mail hadden ontvangen, maar er pas nu aan toekwamen.
17	neutral	Iedereen was zijn CV aan het opstellen, waaronder enkele vrouwen die al een jaar een baan hadden, maar nu op zoek waren naar iets anders.
18	neutral	Iedereen was zijn patiënten aan het behandelen, waaronder enkele vrouwen die al een dubbele dienst erop hadden zitten, maar gelukkig bijna naar huis mochten.
19	neutral	Iedereen was zijn veters aan het strikken, waaronder enkele vrouwen die al tien minuten geleden hadden moeten vertrekken, maar zich hadden verslapen.
20	neutral	Iedereen was zijn ski's aan het aandoen, waaronder enkele vrouwen die al vaker op wintersport waren geweest, maar tot nu toe alleen maar gesnowboard hadden.
21	neutral	Iedereen was zijn koffie aan het opdrinken, waaronder enkele vrouwen die al een half uur pauze hadden gehad, maar nog steeds niet terug aan het werk wilden.
22	neutral	Iedereen was zijn laptop aan het opstarten, waaronder enkele vrouwen die al lang een nieuwe nodig hadden, maar er geen geld voor hadden.
23	neutral	Iedereen was zijn werkmails aan het beantwoorden, waaronder enkele vrouwen die al twee weken vakantie hadden, maar het werk toch niet konden laten liggen.
24	neutral	Iedereen was zijn fiets aan het stallen, waaronder enkele vrouwen die al tien minuten naar een plek hadden gezocht, maar er pas nu een hadden gevonden.
25	male	Iedereen was zijn dak aan het repareren, waaronder enkele vrouwen die al sinds de dag van de storm in de weer waren met emmers, maar het probleem nu grondig wilden aanpakken.
26	male	Iedereen was zijn pijp aan het roken, waaronder enkele vrouwen die al jaren niet meer gerookt hadden, maar nu toch weer waren begonnen.
27	male	Iedereen was zijn auto aan het repareren, waaronder enkele vrouwen die al eerder naar de motor hadden gekeken, maar het probleem nog niet hadden gevonden.
28	male	Iedereen was zijn bouwklus aan het afronden, waaronder enkele vrouwen die al drie maanden bezig waren, maar meteen in het begin vertraging hadden opgelopen.
29	male	Iedereen was zijn pak aan het aandoen, waaronder enkele vrouwen die al over vijf minuten weg moesten, maar dit waarschijnlijk niet zouden gaan halen.
30	male	Iedereen was zijn sigaar aan het roken, waaronder enkele vrouwen die al jaren probeerden om te stoppen, maar het toch niet konden laten.

31	male	Iedereen was zijn borstspieren aan het trainen, waaronder enkele vrouwen die al meer dan een uur in de sportschool waren, maar de work-out nog steeds niet af hadden.
32	male	Iedereen was zijn autobanden aan het verwisselen, waaronder enkele vrouwen die al een maand te lang met winterbanden rondreden, maar niet eerder tijd hadden gehad om deze te vervangen.
33	male	Iedereen was zijn pistool aan het reinigen, waaronder enkele vrouwen die al een uur op de schietbaan hadden gestaan, maar nu ruimte moesten maken voor anderen.
34	male	Iedereen was zijn voetbaltrucs aan het oefenen, waaronder enkele vrouwen die al tien minuten klaar waren met warmlopen, maar eerst op de rest van het team hadden moeten wachten.
35	male	Iedereen was zijn bouwhelm aan het vastmaken, waaronder enkele vrouwen die al een half jaar aan het verbouwen waren, maar pas nu de nieuwe ramen konden plaatsen.
36	male	Iedereen was zijn oliepeil aan het controleren, waaronder enkele vrouwen die al langer een waarschuwingslichtje zagen branden, maar pas vandaag onder de motorkap keken.
37	male	Iedereen was zijn gereedschap aan het klaarleggen, waaronder enkele vrouwen die al dagen van plan waren om te gaan klussen, maar het steeds hadden uitgesteld.
38	male	Iedereen was zijn motorvakantie aan het plannen, waaronder enkele vrouwen die al vaker in eigen land op reis waren geweest, maar nu eens een ander land wilden verkennen.
39	male	Iedereen was zijn geweer aan het laden, waaronder enkele vrouwen die al van jongs af aan op jacht gingen, maar nog nooit een hert hadden geschoten.
40	male	Iedereen was zijn sportauto aan het parkeren, waaronder enkele vrouwen die al heel lang een parkeerplek hadden gezocht, maar er tot nu toe steeds te laat bij waren geweest.
41	male	Iedereen was zijn bokshandschoenen aan het aandoen, waaronder enkele vrouwen die al over een week een wedstrijd hadden, maar nog flink moesten trainen.
42	male	Iedereen was zijn hengel aan het uitwerpen, waaronder enkele vrouwen die al de hele ochtend aan het vissen waren, maar nog steeds niets gevangen hadden.
43	male	Iedereen was zijn mountainbike aan het afstellen, waaronder enkele vrouwen die al enige tijd gefietst hadden, maar het stuur toch te laag vonden staan.
44	male	Iedereen was zijn voetbalschoenen aan het aandoen, waaronder enkele vrouwen die al over vijf minuten op het veld moesten staan, maar de tijd niet in de gaten hadden gehouden.
45	male	Iedereen was zijn krachtoefeningen aan het doen, waaronder enkele vrouwen die al vaker naar de sportschool waren geweest, maar tot nu toe alleen maar cardio hadden gedaan.
46	male	Iedereen was zijn blikje aan het adten, waaronder enkele vrouwen die al een tijdje op het feestje waren, maar nu pas het eerste biertje dronken.

47	male	Iedereen was zijn inzet aan het verhogen, waaronder enkele vrouwen die al twee uur aan het spelen waren, maar nog geen ronde hadden gewonnen.
48	male	Iedereen was zijn tent aan het opzetten, waaronder enkele vrouwen die al een tijdje bezig waren, maar kennelijk een paar haringen tekortkwamen.
49	female	Iedereen was zijn breiwerk aan het afmaken, waaronder enkele vrouwen die al de hele winter met dezelfde trui bezig waren, maar het inmiddels bijna af hadden.
50	female	Iedereen was zijn wenkbrauwen aan het epileren, waaronder enkele vrouwen die al sinds de puberteit regelmatig haartjes verwijderden, maar het nog steeds heel pijnlijk vonden.
51	female	Iedereen was zijn oorbellen aan het indoen, waaronder enkele vrouwen die al een tijdje stonden te prutsen, maar de sluiting er maar moeilijk op kregen.
52	female	Iedereen was zijn balletschoenen aan het aantrekken, waaronder enkele vrouwen die al twee keer hadden opgetreden vandaag, maar nu zelfs nog een derde voorstelling hadden.
53	female	Iedereen was zijn naam aan het borduren, waaronder enkele vrouwen die al een uur bezig waren, maar deze oefening voor beginners nog niet af hadden.
54	female	Iedereen was zijn sieraden aan het opbergen, waaronder enkele vrouwen die al jaren niet meer de moeite daartoe hadden genomen, maar na recente inbraken waren geschrokken.
55	female	Iedereen was zijn roddelblad aan het lezen, waaronder enkele vrouwen die al drie kwartier in de wachtkamer zaten, maar nog steeds niet aan de beurt waren.
56	female	Iedereen was zijn naaimachine aan het klaarzetten, waaronder enkele vrouwen die al een tijdje hadden uitgekeken naar de cursus, maar er de eerste keer helaas niet bij konden zijn.
57	female	Iedereen was zijn pirouettes aan het oefenen, waaronder enkele vrouwen die al vijf jaar op dansles zaten, maar pas binnenkort een eerste uitvoering zouden hebben.
58	female	Iedereen was zijn haar aan het verven, waaronder enkele vrouwen die al vroeg grijs waren geworden, maar niet wilden dat andere mensen dit zagen.
59	female	Iedereen was zijn cupcakes aan het versieren, waaronder enkele vrouwen die al eerder vormpjes van marsepein hadden gemaakt, maar hadden moeten wachten totdat de baksels waren afgekoeld.
60	female	Iedereen was zijn oksels aan het scheren, waaronder enkele vrouwen die al drie weken geen scheermesje hadden aangeraakt, maar zich nu gereedmaakten voor het zwembad.
61	female	Iedereen was zijn dagcrème aan het aanbrengen, waaronder enkele vrouwen die al een uur geleden hadden gedoucht, maar tussendoor eerst hadden ontbeten.
62	female	Iedereen was zijn dagboek aan het bijhouden, waaronder enkele vrouwen die al sinds de basisschool regelmatig schreven, maar er niet meer elke dag aan toekwamen.

63	female	Iedereen was zijn paard aan het borstelen, waaronder enkele vrouwen die al over een uur moesten starten, maar nog lang niet klaar waren met de voorbereidingen.
64	female	Iedereen was zijn yogaoefeningen aan het doen, waaronder enkele vrouwen die al drie weken niet naar de les waren geweest, maar wel thuis hadden geoefend.
65	female	Iedereen was zijn horoscoop aan het lezen, waaronder enkele vrouwen die al jaren elke zondag naar deze rubriek keken, maar de verhalen toch niet geloofwaardig vonden.
66	female	Iedereen was zijn calorieën aan het opschrijven, waaronder enkele vrouwen die al weken op dieet waren, maar nog niet veel waren afgevallen.
67	female	Iedereen was zijn hart aan het uitstorten, waaronder enkele vrouwen die al jaren een vaste relatie hadden, maar nu geen passie meer voelden.
68	female	Iedereen was zijn kaarsen aan het aansteken, waaronder enkele vrouwen die al snel een mooie sfeer hadden gecreëerd, maar toch nog wat meer lichtjes wilden.
69	female	Iedereen was zijn relatieproblemen aan het bespreken, waaronder enkele vrouwen die al lang niet meer gelukkig waren, maar nog niet de moed hadden gehad om het uit te maken.
70	female	Iedereen was zijn thee aan het drinken, waaronder enkele vrouwen die al bij het ontbijt een hele pot hadden gehad, maar nu wel nog een kopje lustten.
71	female	Iedereen was zijn outfit aan het samenstellen, waaronder enkele vrouwen die al een half uur voor de spiegel hadden gestaan, maar nog steeds geen keuze hadden gemaakt.
72	female	Iedereen was zijn was aan het doen, waaronder enkele vrouwen die al een tijdje in de wasserette op een vrije droger zaten te wachten, maar nu eindelijk aan de beurt waren.
