# Supplementary material for: The male bias of a generically-intended masculine pronoun: Evidence from eye-tracking and sentence evaluation
Source: PLoS One. 2021 Apr 1;16(4):e0249309. doi: 10.1371/journal.pone.0249309 (PMC8016286; doi:10.1371/journal.pone.0249309)
Supplement: S1 Pre-tests — (PDF) [file pone.0249309.s004.pdf]

## S1 Pre-tests

*Pre-test 1: Plausibility*

The goal of this pre-test was to test the plausibility of the scenarios described in all potential stimuli and to select those with the highest plausibility rating for the eye-tracking experiment. Put differently, we wanted to avoid that some stimuli described situations which were deemed unlikely – as opposed to others – and this in turn leading to increased reading times.

Twenty-four native Dutch speakers (three male) completed the online questionnaire. They ranged in age from 18 to 29 ($M$ = 21.1). Nineteen participants could be recruited from the Radboud Research Participation System SONA and received credit for participation. The remaining five participants had responded to a participant call on *Facebook* and did not receive any reimbursement.

In order for the pronoun *zijn* 'his' not to bias participants, all stimuli were presented in the control condition. Furthermore, stimuli featuring male stereotype contexts made reference to *a few men* performing the action, while stimuli featuring female stereotype contexts referred to *a few women* performing the action. This was done to make sure that participants rated the context itself and not a potential gender mismatch. Half of the stimuli with neutral stereotype contexts featured male referents and female referents, respectively. Participants were evenly distributed across two lists, which were created so that each potential neutral stimulus occurred with a female and a male continuation half of the time. This design would allow to adapt or exclude neutral stimuli from the eye-tracking experiment for which a female or male referent was perceived as more plausible regardless of the experimental manipulation.

In addition, 30 control items were created, which were intentionally implausible. Half of the controls featured stereotypically female contexts with female continuations and stereotypically male contexts with male continuations, respectively. The stereotype contexts were taken from the rating study above, but were different from the contexts used in the experimental stimuli. All items had to be rated on a 7-point Likert scale. Participants were instructed to indicate for each stimulus how likely they thought the described situation was to occur. *1* stood for *very unlikely*, while *7* stood for *very likely*. The pre-test was administered through Qualtrics (2018).

### Results and selection of experimental stimuli

The average rating of the neutral stimuli ranged between 4.38 ($SD$ = 1.91) and 5.96 ($SD$ = 1). Out of the 32 potential neutral stimuli, the 24 best were selected for the eye-tracking experiment. These 24 neutral stimuli all had received an overall average plausibility rating higher than 4.5. Furthermore, the mean plausibility of all chosen stimuli considering only female continuations or only male continuations exceeded 4.5 as well. The only exception is the stimulus featuring the activity *tanden poetsen* 'brushing teeth', which only received a rating of 4 when featuring the female continuation, but a rating of 5.08 when featuring the male continuation. The sentence refers to men or women who had not been to the dentist *in years*. This was thought to be the source of the generally low rating, as well as the even lower rating for the stimulus variant featuring women, and was therefore changed to *one year* - an arguably more plausible scenario.

Stereotypically female and male contexts were only presented with gender-congruent continuations. The average rating for male stimuli ranged from 4.2 ($SD$ = 1.47) to 6.13 ($SD$ = 1.12). For female stimuli, ratings ranged from 4.25 ($SD$ = 1.98) to 6.42 ($SD$ = 0.76). As with the neutral contexts, 24 out of the 32

potential male and 24 out of the potential female stimuli with an average plausibility rating above 4.5 were chosen for the eye-tracking experiment. The overall item means can be seen in the table below.

Table 1. Mean plausibility rating, standard deviation and range per stereotype context for all 96 possible stimuli and the final selection of 72 stimuli with highest ratings.

| | **Data set** | | | | | |
|---|---|---|---|---|---|---|
| | *96 stimuli* | | | *72 stimuli* | | |
| **Stereotype** | *M* | *SD* | range | *M* | *SD* | range |
| *female* | 5.42 | 1.44 | [4.25; 6.42] | 5.49 | 1.40 | [4.67; 6.42] |
| *male* | 5.24 | 1.48 | [4.21; 6.13] | 5.36 | 1.47 | [4.54; 6.13] |
| *neutral* | 5.39 | 1.45 | [4.38; 5.96] | 5.52 | 1.36 | [4.54; 5.96] |

As intended, the controls were judged as less plausible than the (potential) stimuli and stimulated participants to use the low extreme of the scale, with ratings ranging from 1.13 ($SD$ = 0.45) to 3.04 ($SD$ = 1.83).

*Pre-test 2: Membership*

The aim of this pre-test was to determine whether the noun phrases *enkele mannen* 'some men' and *enkele vrouwen* 'some women' would actually be interpreted as being part of the group previously introduced by *iedereen* 'everyone'. If this were not the case, no valid conclusions regarding the effect of the pronoun *zijn* 'his' could be drawn based on the eye-tracking data. Thus, as the pronoun *zijn* 'his' is introduced in reference to *iedereen* 'everyone', the pronoun would not be interpreted as relating to the men or women referred to in the second sentence *if* a membership reading had not been established. Therefore, the grammatically masculine gender of *zijn* 'his' would not be expected to affect the processing of the noun phrases *enkele mannen* 'some men' and *enkele vrouwen* 'some women'. We tested three possible connectives: *zo ook* 'also' (literally 'so also') versus *waaronder* 'among whom' versus *net als* 'just like'. *Net als* 'just like' served as a baseline as its semantics should evoke a membership reading only to a limited extent, but introduce a new set of people instead.

Twenty-four participants (four male) completed this online pre-test. They were between 18 and 22 years old ($M$ = 19.8). Participants were recruited through the Radboud Research Participation System SONA and received credit for participation.

The pre-test was carried out with 32 potential stimuli (24 of which would be used in the eye-tracking experiment), which were all neutral to make sure that the participants' ratings are not influenced by their knowledge of stereotypes. The possessive pronouns *zijn* and *hun* were removed from the stimuli and – depending on what sounded natural – a determiner was inserted instead, or the position was left unfilled. This was to make sure that the membership reading would not be influenced by the masculine gender of the pronoun *zijn* 'his'. Several characteristics of the stimuli were varied, resulting in four lists; across all lists, twelve of the stimuli featured the connective *zo ook* 'also' , twelve featured *waaronder* 'among whom' and eight featured *net als* 'just like'. For each participant, half the sentences were presented in the experimental condition, in which the group was introduced by *iedereen* 'everyone', while the other half was presented in the control condition, in which the group was introduced by *ze* 'they'.

The second pre-test was also administered through Qualtrics (2018). Participants were asked to rate the 32 items on a 7-point Likert scale and to indicate their interpretation of each stimulus: is the group of women or men mentioned later on in the sentence part of the group mentioned at the very beginning? *1* meant that they surely were not part of this group, *7* meant that they surely were part of this group.

Table 2 shows the results, based on which we were confident that a membership reading could best be achieved by means of *waaronder* ‘among whom’.

Table 2. Mean membership ratings and standard deviations per connective.

| **Connective** | ***M*** | ***SD*** |
|---|---|---|
| *net als* | 4.93 | 1.98 |
| *zo ook* | 5.29 | 1.67 |
| *waaronder* | 5.71 | 1.27 |
