# Supplementary material for: The male bias of a generically-intended masculine pronoun: Evidence from eye-tracking and sentence evaluation
Source: PLoS One. 2021 Apr 1;16(4):e0249309. doi: 10.1371/journal.pone.0249309 (PMC8016286; doi:10.1371/journal.pone.0249309)
Supplement: S1 Model summaries Experiment 1 — (PDF) [file pone.0249309.s005.pdf]

S1 Model summaries Experiment 1.

Fixed-effect coefficients *β*, their *t*-scores, uncorrected *p*-values and the FDR threshold for the fixed effect in question. *P*-values which fall below the FDR threshold are in bold.

**Region 1: Quantifier - first run dwell time**				
	β	*t*	*p*	FDR threshold
Intercept	5.37	395.66	**<0.001**	0.050
Pronoun	0.01	0.42	0.678	0.006
Continuation	0.00	0.37	0.710	0.006
Participant gender	-0.03	-1.05	0.296	0.006
Pronoun * Continuation	-0.04	-1.51	0.131	0.006
Pronoun * Participant gender	0.04	1.23	0.220	0.006
Continuation * Participant gender	-0.03	-1.11	0.279	0.006
Pronoun * Continuation * Participant gender	0.16	3.07	**0.002**	0.006

**Region 1: Quantifier - regression path duration**				
	β	*t*	*p*	FDR threshold
Intercept	5.53	320.90	**<0.001**	0.050
Pronoun	0.03	1.53	0.140	0.006
Continuation	0.01	0.71	0.478	0.006
Participant gender	-0.07	-1.97	0.052	0.006
Pronoun * Continuation	-0.04	-1.06	0.292	0.006
Pronoun * Participant gender	0.02	0.40	0.690	0.006
Continuation * Participant gender	-0.05	-1.26	0.222	0.006
Pronoun * Continuation * Participant gender	0.17	2.06	0.042	0.006

**Region 1: Quantifier - dwell time**				
	β	*t*	*p*	FDR threshold
Intercept	5.56	327.62	**<0.001**	0.050
Pronoun	0.01	0.38	0.705	0.006
Continuation	0.00	-0.01	0.990	0.006
Participant gender	-0.12	-3.41	**0.001**	0.006
Pronoun * Continuation	0.06	1.54	0.125	0.006
Pronoun * Participant gender	0.01	0.21	0.838	0.006
Continuation * Participant gender	-0.10	-2.50	0.014	0.006
Pronoun * Continuation * Participant gender	0.08	1.05	0.294	0.006

**Region 2: Noun - first run dwell time**				
	β	*t*	*p*	FDR threshold
Intercept	5.27	403.57	**<0.001**	0.050
Pronoun	0.02	1.44	0.149	0.006
Continuation	-0.04	-2.13	0.043	0.006
Participant gender	-0.05	-1.96	0.053	0.006
Pronoun * Continuation	-0.02	-0.64	0.528	0.006
Pronoun * Participant gender	0.00	0.02	0.982	0.006
Continuation * Participant gender	-0.02	-0.49	0.627	0.006
Pronoun * Continuation * Participant gender	0.03	0.49	0.624	0.006

**Region 2: Noun - regression path duration**				
	β	*t*	*p*	FDR threshold
Intercept	5.38	255.91	**<0.001**	0.050
Pronoun	0.03	1.40	0.161	0.006
Continuation	-0.04	-1.61	0.111	0.006
Participant gender	-0.06	-1.57	0.120	0.006
Pronoun * Continuation	0.03	0.73	0.475	0.006
Pronoun * Participant gender	-0.04	-1.10	0.271	0.006
Continuation * Participant gender	-0.08	-1.51	0.142	0.006
Pronoun * Continuation * Participant gender	-0.05	-0.60	0.551	0.006

**Region 2: Noun - dwell time**				
	β	*t*	*p*	FDR threshold
Intercept	5.42	284.89	**<0.001**	0.050
Pronoun	0.00	-0.12	0.904	0.006
Continuation	-0.02	-0.69	0.494	0.006
Participant gender	-0.09	-2.55	0.013	0.006
Pronoun * Continuation	-0.01	-0.13	0.897	0.006
Pronoun * Participant gender	0.00	0.05	0.960	0.006
Continuation * Participant gender	-0.04	-0.74	0.467	0.006
Pronoun * Continuation * Participant gender	-0.04	-0.50	0.620	0.006

**Region 3: Spillover - first run dwell time**				
	β	*t*	*p*	FDR threshold
Intercept	5.40	359.54	**<0.001**	0.050
Pronoun	0.02	1.15	0.263	0.006
Continuation	0.03	1.55	0.137	0.006
Participant gender	-0.01	-0.43	0.667	0.006
Pronoun * Continuation	-0.04	-1.17	0.245	0.006
Pronoun * Participant gender	0.03	0.99	0.324	0.006
Continuation * Participant gender	-0.04	-1.41	0.163	0.006
Pronoun * Continuation * Participant gender	-0.03	-0.33	0.741	0.006

**Region 3: Spillover - regression path duration**				
	β	*t*	*p*	FDR threshold
Intercept	5.47	331.77	**<0.001**	0.050
Pronoun	0.01	0.54	0.597	0.006
Continuation	0.03	1.42	0.167	0.006
Participant gender	-0.02	-0.51	0.611	0.006
Pronoun * Continuation	-0.03	-0.87	0.386	0.006
Pronoun * Participant gender	0.01	0.25	0.802	0.006
Continuation * Participant gender	-0.08	-1.93	0.054	0.006
Pronoun * Continuation * Participant gender	-0.02	-0.26	0.797	0.006

**Region 3: Spillover - dwell time**				
	β	*t*	*p*	FDR threshold
Intercept	5.55	287.58	**<0.001**	0.050
Pronoun	0.02	1.12	0.267	0.006
Continuation	0.02	0.65	0.520	0.006
Participant gender	-0.07	-1.93	0.056	0.006

Pronoun * Continuation	0.00	-0.06	0.950	0.006
Pronoun * Participant gender	0.01	0.21	0.835	0.006
Continuation * Participant gender	-0.01	-0.32	0.753	0.006
Pronoun * Continuation * Participant gender	0.03	0.25	0.805	0.006

Extended analysis to all stereotype contexts for male participants only. Fixed-effect coefficients *β*, their *t*-scores, uncorrected *p*-values and the FDR threshold for the fixed effect in question. The FDR threshold was calculated by ordering the *p*-values of the three effects of interest (shown in bold). *P*-values which fall below the FDR threshold are in bold.

**Region 1: Quantifier - first run dwell time**				
	β	*t*	*p*	FDR threshold
Intercept	5.38	293.58	**<0.001**	NA
Pronoun	0.002	0.15	0.884	NA
Continuation	0.01	0.47	0.642	NA
Stereotype (*female* vs. *neutral*)	-0.01	-0.48	0.634	NA
Stereotype (*male* vs. *neutral*)	-0.002	-0.14	0.891	NA
**Pronoun * Continuation**	-0.03	-1.07	0.285	0.017
Pronoun * Stereotype (*female* vs. *neutral*)	0.01	0.27	0.786	NA
Pronoun * Stereotype (*male* vs. *neutral*)	0.03	0.77	0.443	NA
Continuation * Stereotype (*female* vs. *neutral*)	-0.06	-1.89	0.059	NA
Continuation * Stereotype (*male* vs. *neutral*)	0.01	0.46	0.644	NA
**Pronoun * Continuation * Stereotype (*female* vs. *neutral*)**	0.15	2.37	0.018	0.017
**Pronoun * Continuation * Stereotype (*male* vs. *neutral*)**	0.12	1.91	0.059	0.017
