# Supplementary material for: The male bias of a generically-intended masculine pronoun: Evidence from eye-tracking and sentence evaluation
Source: PLoS One. 2021 Apr 1;16(4):e0249309. doi: 10.1371/journal.pone.0249309 (PMC8016286; doi:10.1371/journal.pone.0249309)
Supplement: S2 Model summaries Experiment 2 — (PDF) [file pone.0249309.s006.pdf]

S2 Model summaries Experiment 2.

Fixed-effect coefficients *β*, their *t*-scores and *p*-values shown for response type and response time.

**Response**			
	β	*z*	*p*
Intercept	2.90	17.07	**<0.001**
Stereotype (*female* vs. *neutral*)	-0.24	-1.25	0.210
Stereotype (*male* vs. *neutral*)	-0.36	-1.87	0.061
Continuation	-0.02	-0.10	0.921
Participant gender	0.38	1.24	0.214
Continuation * Stereotype (*female* vs. *neutral*)	0.11	0.36	0.718
Continuation * Stereotype (*male* vs. *neutral*)	-0.06	-0.20	0.845
Stereotype (*female* vs. *neutral*) * Participant gender	0.27	1.05	0.296
Stereotype (*male* vs. *neutral*) * Participant gender	0.26	1.04	0.297
Continuation * Participant gender	0.17	0.49	0.626
Continuation * Stereotype (*female* vs. *neutral*) * Participant gender	-0.22	-0.45	0.655
Continuation * Stereotype (*male* vs. *neutral*) * Participant gender	-0.39	-0.78	0.434

**Response time**			
	β	*t*	*p*
Intercept	7.72	208.75	**<0.001**
Stereotype (*female* vs. *neutral*)	-0.03	-0.94	0.351
Stereotype (*male* vs. *neutral*)	-0.04	-1.21	0.230
Continuation	0.00	0.12	0.905
Participant gender	0.03	0.47	0.642
Continuation * Stereotype (*female* vs. *neutral*)	-0.05	-1.83	0.072
Continuation * Stereotype (*male* vs. *neutral*)	0.02	0.78	0.435
Stereotype (*female* vs. *neutral*) * Participant gender	0.00	0.16	0.873
Stereotype (*male* vs. *neutral*) * Participant gender	0.02	0.74	0.460
Continuation * Participant gender	-0.04	-1.84	0.066
Continuation * Stereotype (*female* vs. *neutral*) * Participant gender	0.01	0.20	0.840
Continuation * Stereotype (*male* vs. *neutral*) * Participant gender	0.00	-0.05	0.961
