# Supplementary material for: The male bias of a generically-intended masculine pronoun: Evidence from eye-tracking and sentence evaluation
Source: PLoS One. 2021 Apr 1;16(4):e0249309. doi: 10.1371/journal.pone.0249309 (PMC8016286; doi:10.1371/journal.pone.0249309)
Supplement: S1 Exploratory analyses — (PDF) [file pone.0249309.s008.pdf]

S1 Exploratory analyses

1. *Regressions to first clause (yes/no)*

We modelled whether regressions back to the first clause occurred (i.e., *Iedereen was zijn veters aan het strikken* 'Everyone was tying his shoelaces' and *Ze waren allemaal hun veters aan het strikken* 'They were all tying their shoelaces'). We fitted a mixed effects logistic regression model from the binomial family using the *glmer* function from the *lme4* package, using the same model simplification approach as described in the main paper. No significant effects relevant to the hypothesis were found.

	ß	*t*	*p*
Intercept	-4.47	-14.52	<0.001
Pronoun	-0.03	-0.10	0.920
Continuation	0.20	0.74	0.458
Participant gender	-0.89	-2.36	0.018
Pronoun * Continuation	0.16	0.29	0.771
Pronoun * Participant gender	0.08	0.14	0.888
Continuation * Participant gender	-0.26	-0.47	0.636
Pronoun * Continuation * Participant gender	-1.31	-1.32	0.187

2. *Number of regressions launched from pre-view region*

We analyzed the total number of regression fixations starting at the quantifier region (i.e., the region in which the male bias was found for first run dwell time) to any region before it. Note that this then includes regressions to the entire first clause as well as to *waaronder* 'among whom'. We fitted a generalized mixed effects model from the poisson family with a log link function using the *glmer* function from the *lme4* package. No significant effects were found.

	ß	*t*	*p*
Intercept	-2.24	-21.14	<0.001
Pronoun	0.12	1.04	0.300
Continuation	0.07	0.56	0.575
Participant gender	-0.16	-0.87	0.382
Pronoun * Continuation	0.07	0.31	0.753
Pronoun * Participant gender	0.02	0.08	0.935
Continuation * Participant gender	-0.10	-0.44	0.658
Pronoun * Continuation * Participant gender	-0.11	-0.23	0.820
