# Supplementary material for: The male bias of a generically-intended masculine pronoun: Evidence from eye-tracking and sentence evaluation
Source: PLoS One. 2021 Apr 1;16(4):e0249309. doi: 10.1371/journal.pone.0249309 (PMC8016286; doi:10.1371/journal.pone.0249309)
Supplement: Attachment — Submitted filename: Response to reviewers - .docx [file pone.0249309.s009.docx]

Editor’s comments:

*Your manuscript has been evaluated by two reviewers, who find your manuscript to be well written and*

*its topic interesting and timely. Between them they also raise several points that will need to be*

*addressed before the manuscript can be reconsidered for publication, however. One specific concern*

*is the introduction, which they suggest should be revised so as to include more information about the*

*Dutch gender system, an improved overview of the literature on context effects on pronoun*

*interpretation, and a proper introduction of the "gender of participant hypothesis" that your study*

*investigates. The reviewers also ask you to improve the description of experimental design and motivate*

*your choice of interest regions. I urge you to pay particular attention to the concern about a potential*

*bias in your experimental materials, due to your stimulus items being introduced by the indefinite*

*pronoun iedereen ‘everyone’, which might have biased participants towards a generic reading of the*

*pronoun 'zijn'. Both reviewers suggest that your discussion be revised, to better explain the unexpected*

*absence of a male bias in the male stereotyped condition and in the light of potential biases in your*

*materials. Please also attend carefully to the reviewers' minor comments.*

We addressed all these points raised (i.e., review of Dutch gender system, context effects on pronoun

interpretation, participant gender effect, design with 12 conditions, chosen eye-tracking measures and

regions, materials, absence of effect in male stereotype condition) in detail in response to the feedback

provided by Reviewer 1 and Reviewer 2 below.

Updated cover letter including the response to reviewers

*Reviewer #1*

*Main concerns:*

*1. My main concern is about the materials, i.e., the sentences that are used. Note that I don’t know*

*Dutch at all, so I am relying on the English translations. Now, I am concerned that the first parts of the*

*sentences, despite the pronoun in the masculine form, are actually priming inclusion. Namely, they use*

*“everyone” as the reference of the pronoun. So really, this primes “his” to be generically understood.*

*A little bit like in Gygax et al. (2012) when participants were explicitly asked to consider the masculine*

*form generically (which resulted in more inclusive representations, although still masculine). Past*

*research has been concerned with generic and specific interpretations of the masculine form when NO*

*context is given (except stereotyped ones). As such, it is really not that surprising that people can see*

*the masculine form as inclusive, especially that the first word of the sentence actually states that it IS*

*inclusive.*

*I am therefore wondering whether in sentences like these, the pronoun would be treated differently:*

*When tying his shoes, the women seemed stressed.*

*[Terwijl hij zijn schoenen strikte, leek de vrouw gestrest.]*

*Or, if this is actually grammatically wrong:*

*When the musician tied his shoes, she seemed stressed.*

*[Terwijl de muzikant zijn schoenen strikte, leek zij gestrest.]*

*or*

*When a musician prepares his guitar, she is always concentrated.*

*[Wanneer een muzikant zijn gitaar voorbereid, ze is altijd geconcentreerd.]*

*In Experiment 2, this is even increased by the inclusion of “Someone” in the filler items. So the filler*

*items are about an individual, whereas the experimental items are about “Everyone”. In fact, the*

*difference between the fillers and the experimental items could explain why there was no effect in*

*Experiment 2.*

Designing the materials in this way including *iedereen* ‘everyone’ was a conscious choice on which we

elaborate below as well as in the manuscript.

The main reason for choosing an indefinite pronoun (i.e., *iedereen* ‘everyone’) over any of the example

stimuli provided by Reviewer 1 is to isolate the effect of the pronoun. We know that masculine role

nouns lead to a male bias when used generically (also in Dutch: Backer & Cuypere, 2012; Vervecken

& Hannover, 2015), thus using a role noun as the antecedent for the pronoun *zijn* ‘his’ would strongly

bias the materials towards our H1 (i.e., that *zijn* ‘his’ leads to a male bias). If we then find evidence of

a male bias, it would not be clear whether this is only due to the role noun or whether the pronoun also

played a role. Thus, using a role noun as an antecedent introduces a confound. Consider one of the

stimuli that Reviewer 1 suggests:

When a musician prepares his guitar, she is always concentrated.

[Wanneer een muzikant zijn gitaar voorbereid, is ze altijd geconcentreerd.]

If one were to find a reading time increase on *she*, it would be entirely unclear whether this male bias

is caused by the pronoun, by the role noun or both. This therefore excludes role nouns as antecedents

when testing pronouns, at least in Dutch and other languages in which they are marked for grammatical

gender.

Crucially, it is also a necessity for the purpose of our experiment to embed *zijn* ‘his’ in contexts in which

the generic reading is intended and in theory possible. Put differently, in order to test whether a

masculine form can successfully be interpreted as generic, one has to provide a context in which this is

at least a possibility. Crucially, previous experiments on role nouns did exactly that. For example,

Gygax et al. (2008) used sentences such as the following:

*Die Nachbarn liefen durch den Bahnhof.*

‚The neighbors (MASC.) were walking through the station’

Masculine role nouns are frequently used generically in plural contexts and Gygax et al. (2008) provide

such a context. As Gygax et al. (2008) say themselves, the noun phrase *die Nachbarn* ‘the neighbors

(MASC.) is ambiguous between a masculine reading (i.e., the neighbors are only men) or a generically

intended reading (i.e., the neighbors are of mixed or possibly even only female gender). The goal of

their experiment was to determine whether this ambiguity is resolved to women’s disadvantage.

Similarly, *zijn* ‘his’ can be used to refer to men only, but it is also used to refer to all genders in certain

contexts; similar to Gygax et al. (2008) and many other experiments on role nouns, our experiment

tested how this ambiguity is resolved.

This ties in with another issue that Reviewer 1 raises. Reviewer 1 suggests that previous experiments

tested masculine generics when *no* context was given, and that our experiments violates this. We argue

that this is not the case, as already hinted at above. For example, previous experiments on role nouns as

masculine generics have tested plural contexts (e.g., Garnham et al., 2012; Gygax et al., 2008) while

others have tested singular contexts (e.g., Irmen & Schumann, 2011). De Backer and De Cuypere (2012)

have shown that singular role nouns in Dutch and German have a stronger male bias than plural role

nouns. Thus, whether materials make use of singular or plural contexts affects the outcome. In fact, this

is something we confirmed for *zijn* in a follow-up experiment (currently under review elsewhere) in

which we compared conceptually singular and plural stimuli. To give another example, it likely makes

a difference whether materials use episodic contexts (i.e., referring to a contextually determined group

of people like in the current study or in Gygax et al. and Garnham et al.) or whether the materials are

truly generic (i.e., generalizing over people, like in the stimuli used by Irmen, 2007). Thus, it is truly

not the case that all previous experiments on role nouns provided no context, while ours does.

Take the stimulus suggested by Reviewer 1 again:

When a musician prepares his guitar, she is always concentrated.

[Wanneer een muzikant zijn gitaar voorbereid, is ze altijd geconcentreerd.]

This sentence provides a generic context in the semantic sense: it generalize over *all* musicians and

makes a statement about them. Our stimuli, on the other hand, are referential (as opposed to generic)

and refer to a contextually determined group of people. As Irmen (2007, p432) writes: “Non-referential

role names denote abstract concepts rather than concrete persons (Teachers often say that…vs. The

teacher often said that…)…Thus, for semantic as well as formal reasons it is plausible to assume that

gender information is less relevant in generic than in referential contexts.” Thus, the proposed stimulus

potentially biases readers towards a generic reading more than the referential stimuli that we used.

For the sake of completeness, we would like to comment on the other stimuli suggested by Reviewer 1

as well:

*When tying his**i** shoes, the women**i** seemed stressed.

[*Terwijl hij**i** zijn**i** schoenen strikte, leken de vrouwen**i** gestrest.]

A reading in which *hij* ‘he’, *zijn* ‘his’ and *vrouwen* ‘women’ refer to the same person would be

ungrammatical and would therefore not arise. Thus, this is a context in which language users would

never use *zijn* generically – since it is in fact ungrammatical. It is therefore not suited to test whether

*zijn* can function as a generic. As mentioned above, a context that is ambiguous between a male-specific

and a generic reading of *zijn* ‘his’ is necessary for that.

*When the musiciani tied hisi shoes, shei seemed stressed.

*Terwijl de muzikanti zijni schoenen strikte, leek ziji gestrest.

*His* and *musician* cannot be coreferential with *she* here. This reading is likewise ungrammatical.

And to address the final remark of Reviewer 1 regarding the fillers in Experiment 2, we would like to

point out the similarities with the stimuli: both are episodic, both make use of a quantified pronoun and

both are contexts in which *zijn* ‘his’ can be used generically (*Iedereen/iemand was zijn tanden aan het*

*poetsen* ‘Everyone/someone was brushing his teeth’). We thus consider it unlikely that the fillers are

the reason no effect was found in Experiment 2.

Thus, ultimately, we are convinced that our stimulus materials were adequate for the research question.

If anything, we biased the stimuli against our H1 (which was that there would be a male bias).

We added a motivation of the choice for *iedereen* under the heading “Experiment 1: Eye-tracking during

reading”, right before the changes we made to the Redl et al. (2018) design are discussed.

*2. My second concern is about the regions of interest chosen. I did not understand why the pronoun*

*“zijn” was not also a region of interest. I mean, not necessarily for first run (although this could inform*

*us as to the pre-processing), but mainly as regression fixations. I would imagine that if the system comes*

*across a female mention in the second part of the sentence, whereas it was introduced by a masculine*

*pronoun, it would re-check whether it was really a masculine form. It could even want to check the*

*“everyone” mention (see my main issue). To be on the safe side, I would actually take the whole first*

*part (until the comma), as region of interest. To me, this is very important, especially when looking at*

*regressions in the “their” condition (which I would guess are pretty absent). I think I would use number*

*of regression fixations.*

This is a valid point and we have also considered looking into regressions to the pronoun (or other

regions in the first clause) in more detail when planning the analysis. Let me elaborate on our initial

reasoning regarding the decision not to do so. First and foremost, we had decided against it since we

had already included regression path duration on the three regions we analyzed. Like Reviewer 1, we

thought it possible that readers would refixate the first clause upon encountering a female mention after

a masculine pronoun, and regression path duration was expected to cover this. This is a measure of

duration rather than count as Reviewer 1 suggests, but the two are of course highly correlated, and we

did not expect to necessarily gain additional insights by investigating the regression count into the

pronoun region rather than regression duration out of the regions of interest in the second clause.

Speaking in statistical terms, we did not include any additional measures in order to balance the Type I

and II error rate. The more regions and measures one analyzes, the more likely it is that a significant

result is actually a Type I error. We corrected for this using the false discovery rate control by Benjamini

and Hochberg (1995; in our case, this was very similar to a Bonferroni correction). However, including

more measures/regions while correcting for an inflated Type I error rate also boosts the chance of a

Type II error: the more regions/measures one adds while correcting for it, the smaller the p-value has

to be in order to be considered significant. A meaningful effect might very well go unnoticed as a Type

II error.

Based on Reviewer 1’s comments, we extended the analysis after all. Unfortunately, the combination

of measure and region that Reviewer 1 suggests is not possible, because all reading time measures were

calculated separately for every single word in the first clause. We conceived of several alternatives that

would achieve a similar goal:

1) We calculated whether the entire first clause was fixated as part of a regression (yes/no). There

were no significant effects relevant to the research question.

2) As an alternative, we looked at the number of regressions into the pronoun region. This includes

all regressions launched from any region beyond the pronoun region into the pronoun region.

No significant effects were found.

3) As yet another alternative, we also analyzed the total number of regression fixations starting at

the quantifier region (i.e., the region in which the male bias was found for first run dwell time)

to any region before it. Note that this then includes regressions to the entire first clause as well

as to *waaronder* ‘among whom’. No significant effects were found.

We decided not to include any of these additional analyses in the revised manuscript due to the reasons

provided above, but we are willing to reconsider if the reviewer (or the editor) insists.

*3. I would actually present sets of analyses, but different for each measure (not only locations). To me,*

*each measure illustrate a different cognitive process (and the authors should be clearer as to why they*

*chose those measures, and to which mental processes they refer to). So dwell time, for example, includes*

*a wrap-up process, whereas first path looks at more spontaneous processes. Not quite sure that*

*comparing different models with different measures makes sense.*

We think that this comment is based on a minor misunderstanding. We talk about model comparisons

in the paper regarding the need to correct for the fact that we test the same hypothesis in several regions

and for several measures. This is usually referred to as the *multiple comparisons problem* (or *multiple*

*testing problem*) and can be solved by applying a correction (FDR as we did, or Bonferroni etc.). We

do not actually directly compare the different models to each other. We changed the term *multiple*

*comparisons* to *multiple tests* in the manuscript in order to avoid this misunderstanding.

We further added information on our choice for the reading measures under “Analysis” in Experiment

1:

“We decided to include early as well as late measures of reading, as earlier eye-tracking studies have

shown that both masculine generics and gender stereotypes can affect the reading process from early

to late (Irmen, 2007; Irmen & Schumann, 2011). We opted for the same reading time measures as Redl

et al. (2018), except that we omitted first fixation duration. This was done since previous studies have

either found no reliable effects for first fixation duration (Irmen & Schumann, 2011; Redl et al., 2018)

or found the results for first fixation duration to be similar to those regarding first run dwell time (Irmen,

2007). First run dwell time is regarding as the upper bound of earliest processing, with regression path

duration and dwell time being indicative of later processes (Kliegl & Laubrock, 2017).”

*4. Finally, the fact that no male bias was found for the male stereotyped condition is problematic to me.*

*The fact that the neutral stereotype condition did get it (even only for male participants) makes sense,*

*but in this case, why would the male stereotype condition not have it. This is rather odd. The only*

*difference with the neutral condition is that it increases the sense of maleness. If anything, it should be*

*stronger than the neutral condition… the discussion given by the authors is confusing, as they do not*

*clearly say why they did not find the male bias in the male stereotyped context. …. If one thinks that*

*there are two possible biases, then one of them should lead reading. For the male stereotype context,*

*both male biases should result in the same pattern.*

We understand the confusion and we strove to make that clearer in the discussion of Experiment 1 (see

below). Among other things, we added a reference to an experiment by Gabriel and Gygax (2008),

which found the same result pattern as us (i.e., male bias of the masculine generic word form in neutral

contexts, but gender stereotype overriding grammatical gender in female and male contexts, thereby

not leading to a “double” bias in male stereotype contexts). We also drew parallels to the fact that

researchers (e.g., Gygax et al., 2008) previously found one type of gender cue to fully override the other.

“This result pattern can be explained by assuming that the female and male stereotype contexts overrode

the grammatical information of the pronoun when processing the first sentence clause featuring both

gender cues. Thus, in the case of female and male gender stereotypes, the continuation in the second

clause would then be compared against a mental model based only on stereotypical, but no grammatical

gender information. This idea is supported by the results reported by Gabriel and Gygax [5]. Their

sentence evaluation experiment tested stereotypically male, female and neutral role nouns in

Norwegian. Norwegian is very similar to Dutch regarding the grammatical gender system, as both fall

somewhere in between that of grammatical gender languages such as German and natural gender

languages such as English [5,28]. Gabriel and Gygax found that the grammatical gender information of

Norwegian role nouns led to a male bias, but only when the role nouns were stereotypically neutral. In

the case of stereotypically male or female role nouns, the results were guided by the gender stereotype

and *not* by grammatical gender. Female and male stereotype information thus overrode the grammatical

gender information of the generically-intended role nouns. The authors note that there was a slight

indication that the grammatical gender of the role nouns may have attenuated female gender stereotypes,

but the authors warn to be cautious regarding this interpretation due to a small effect size. However,

when looking at stereotypically *male* contexts, there was absolutely no additional effect of grammatical

gender; the authors conclude that the results were guided by the male gender stereotype alone.

Studies on grammatical gender languages such as French and German suggest that the grammatical

gender information of masculine generics can fully override stereotype information [3,31]. Thus, like

Gabriel and Gygax [5], these authors did not find a “double” male bias when masculine role nouns were

stereotypically male. In German and French, referent gender was inferred based on grammatical gender

alone, as gender stereotypes were overridden by grammatical gender [3,31]. In languages such as

Norwegian and Dutch, however, the opposite seems to be possible: gender stereotype information

seems to be able to override grammatical gender information of (at least some types of) masculine

generics [5]. Thus, the grammatical gender of masculine generics has previously been found to override

gender stereotypes in some languages, but the opposite has been reported for other languages.

If the male and female stereotype contexts indeed overrode the pronoun’s grammatical gender in our

experiment, we would of course expect to see an effect of the stereotype itself when the gendered noun

phrase is encountered. More specifically, we would expect an increase in reading time for noun phrases

of which the gender does not match the gender stereotype (i.e., *women* + male stereotype; *men* + female

stereotype), or at least for male referents in stereotypically female contexts, as Redl et al. [29] had

found. At this early point in processing, we do not observe this. However, Redl et al. only found an

effect of a stereotype violation later on in processing; male protagonists engaging in stereotypically

female activities led to an increase in regression path duration and dwell time on the gendered noun

(i.e., a proper noun in their case). In our current experiment, we solely focused on the effect of the

masculine possessive pronoun and its potential interaction with stereotype information. Therefore, we

did not analyze the female and male gender stereotype contexts when no effect of the pronoun was

found. However, a visual inspection of the later regions and reading times suggests that our data, too,

show this asymmetrical stereotype violation effect later on, namely for dwell time on the gendered noun

(see S6 Figure in the Supplementary Material). Just like previous studies featuring stereotypical

activities [29,61,62], only male protagonists engaging in stereotypically female activities led to a

mismatch effect, but not the other way around. This asymmetry can be explained by the fact that men

are generally penalized more by society for transgressing gender roles [63–66]. Importantly, this does

not suggest that only female gender stereotypes trigger gender inferences [see also 29,61,62]. Thus,

even if the mismatch effect of gender stereotypes is asymmetrical in our experiment, this does in no

way refute the explanation that female and male gender stereotypes overrode the grammatical gender

information of the pronoun *zijn* ‘his’.

*5. It would be nice for readers that do not know Dutch so well to explain in more details the grammatical*

*gender properties in Dutch.*

We have added an introduction to the Dutch gender system with a particular focus on masculine

generics, also citing the Language Index by Gygax et al. (2019).

*6. P3: The authors mention “masculine generics”. Does it mean these are different to masculine*

*specifics? Or are they just masculine forms, used or interpretable as generics? I find the terms*

*“masculine generics” misleading (although I have at times also used it).*

We elaborated on our definition of masculine generics that we provide at the very beginning of the

introduction to make this clearer. We use the term to refer to word forms that are grammatically

masculine, but can be interpreted as male-specific as well as generic.

*7. P3:L9: I’m not sure why it is “clearly”. I don’t see anything in the sentence that would give me such*

*a clear context. At least, to me, it is less clear than if the sentence was introduced by “everyone”, which*

*is the case in the present study.*

We changed this to: “When reading the newspaper article, it becomes clear that the headline is intended

to refer to students regardless of their gender.”

*8. P3:L10: The authors mention that “…has occupied linguists for decades…”. …, I would say that*

*linguists are not the only ones to address these questions (not that a lot of the authors mentioned in the*

*paper are actually psychologists, or psycholinguists):*

We did not intend to give the impression that *only* (psycho)linguists studied this phenomenon, hence

we simply changed it to *researchers*.

*9. P3:L23: a good/sensible continuation. I would add “sensible”.* Changed accordingly.

*10. P3:L26: “therefore taps less into online processing”: This is an old debate about online vs offline*

*processes. I think that this debate is not so informative. Even if you measure eye movements. When*

*making judgements about sentences, i.e., what is considered off-line, people use their mental models to*

*answer, and these mental models were also built on-line. Anyway, I would concentrate on early*

*processes versus later processes. But that’s maybe just my opinion.*

We added mentions to early versus late processing (in addition to online and offline).

*11. P5:L59: the reference should be [15], right?*

Indeed. Thank you for catching this.

*12. P5: While reading, I kept asking myself about what measures the authors used for their eye-tracking.*

*Even when they present Redl et al.’s study, I was wondering what measures they took: dwell time, first*

*fixation, number of fixation, spill over regions,… (it is only mentioned on line 123).*

We are now more explicit about this early on, for example right before example (2):

“They constructed sentences featuring the possessive pronoun *zijn* ‘his’ in generically-intended contexts

similar to the studies on role nouns, and analyzed early as well as late measures of reading on a male or

female proper noun later on, for example:”

And below example (2):

“They did, however, find that the violation of a gender stereotype led to an increase in dwell time (also

called total fixation duration) on the proper noun, but for male protagonists transgressing expectations

based on stereotypes only.”

*13. P6: L94: To my knowledge, except from the study they cite here (and maybe a handfull of others),*

*none of the other studies did find any gender of participant effect. It might be worth mentioning this.*

The study we cited was actually a meta-analysis of 19 studies on masculine generics in English (thus,

mostly pronouns), which found the participant gender effect to be highly robust. We admit that this was

not clear and we thus elaborated on this effect, also already naming a possible reason for this effect in

the introduction. In addition, Redl et al. (2018) had found an interaction effect involving participant

gender, even if it had no bearing on the tested hypothesis. We also included that to make clear why we

thought that adding participant gender (as was also done by Redl et al. (2018)) was important.

*14. P6: L88: The authors seem to say that Redl et al. needed improvement in the design. It would be*

*good to say early what the problems were.*

There was no indication that there were any concrete problems with the design by Redl et al. (2018).

However, there is always room for improvement. To avoid the impression that we thought Redl et al.

(2018) was flawed, we now refer to *changes* and *adaptations* more often than we reference

improvements. We also added the following sentence before going into the changes to make this clearer

(this sentence can be found above Table 2):

“While there was no indication that the male bias of the pronoun was masked by any issues with the

design by Redl et al., we tried to further improve the design wherever possible.”

*16. P8: L145: I am a little puzzled by the gender of participant hypothesis. Of course, it is based on*

*several studies on the pronoun, but I am wondering what would the underlying mechanism be. It could*

*be that as women are exposed to masculine forms when they are referred to (or when they would like*

*to be referred to), they are more used to consider it as generic. Actually, the authors discuss that later*

*on. It could be introduced early to consolidate their hypothesis.* Changed accordingly. See our response

to comment 13 above.

*17. P8: L148: Presenting method before participants is rather odd, right?*

Information on participants indeed usually precedes the method. However, this would force us to make

reference to the exclusion criteria based on the materials before the exclusion criteria could be properly

introduced. This made for very cumbersome reading with Experiment 2, where we used a more intricate

exclusion system based on responses to the materials, because the experiment was run online (and thus

to filter out participants who might have been after easy course credit). It thus made sense to change the

order for Experiment 2 and we then did the same for Experiment 1 in order to be consistent within the

article. Thus, we believe that the paper reads more easily this way.

*19. P17:L82: I think that Gygax, Lévy et al., 2019, found that actually, the male bias was more*

*pronounced for girls (between 3-5 years old)*

We reread the paper mentioned by the reviewer, but could not find the effect that Reviewer 1 refers to.

The authors did not add participant gender to their final model, since the effect was not significant and

therefore no such interaction effect was reported.

*20. P22: L519: good/sensible. Adding “sensible” is important, as in earlier work in English (Garnham,*

*Oakhill, Reynolds), the term “sensible” was used, and in Gygax et al., the French translation was*

*something between good and sensible.*

Changed accordingly.

*21. Depending on the changes made according to the issues raised, I would imagine the discussion to*

*change substantially.*

We have indeed made elaborate changes to the discussion of Experiment 1 in particular, as well as to

the introduction of the paper in order to address all of Reviewer 1’s remarks.

*Reviewer #2*

*I believe that readers would benefit from some more information on the Dutch gender system in the*

*Introduction. The authors mention its peculiarities later on in the Discussion, however, to better*

*understand the research question and the motivation of the study it needs to be explained earlier.*

We added extensive information on the Dutch gender system with a focus on masculine generics.

*I would also like to see a more comprehensive overview of the relevant literature on the effects of context*

*on pronoun interpretation as part of the Introduction (e.g., Duffy & Keir, 2004).*

We extended the introduction to include literature on the effect of (stereotype) contexts on pronoun

interpretation, including the reference mentioned by Reviewer 2.

*Finally, since the participants’ gender is at the core of the main finding, introducing research on*

*differences (or the lack thereof) between men and women in their perception of gender biases would*

*certainly be useful. This could also be used to provide more theoretical grounding for why participants’*

*gender was included in the analysis in the first place.*

We elaborated on this during in the introduction, adding more information on previous literature which

found an effect of participant gender, as well as pointing out one finding by Redl et al. (2018) that also

emphasized the importance of adding participant gender to the design (just like Redl et al. (2018) tested

for it).

*A methodological concern related to the previous comment: the authors state that they “strove to*

*balance their sample between female and male participants”. However, the number of female and male*

*participants is not equal. Since participants’ gender is related to the main finding of the study, this*

*should be justified.*

We added *as much as possible* to the sentence quoted by Reviewer 2. Unfortunately, the answer to this

question is bound to be unsatisfying. These were simply all the male participants we could test. We

tested over the course of a whole academic year, advertised as much as possible, distributed flyers, paid

student assistants to specifically approach all their male friends and acquaintances to motivate them to

sign up for our study, tested until late in the evening so that working participants could also sign up,

advertised in classes etc. We had decided beforehand that the usable data of 48 participants was the

minimum at which we could stop (i.e., 6 per list). This was predominantly based on previous experience

at the lab regarding what number of male participants would be feasible.

*Even though the Experiment 1 is described in great detail, I found it difficult to understand its design.*

*More precisely, it is not quite clear to me why the authors talk about six (rather than one) “control”*

*conditions and why it was necessary to treat them as such in the analyses. Furthermore, how can we be*

*certain that “their” does not potentially carry a male-bias as well? My next comment addresses a*

*related concern.*

We addressed this underneath example (3) and hope this clarifies the issue:

“This allowed us to more clearly identify a possible effect of the masculine pronoun, as the only relevant

difference between the control and the experimental conditions is the pronoun itself. More specifically,

the neutral *control* conditions do not contain *any* gender information in the first clause; by comparing

them to the neutral *experimental* condition, we can make absolutely sure that any found effect is truly

due to the pronoun and not due to a more general male bias (Hamilton, 1991; Irmen, 2007). Thus, if the

difference between the experimental and the control conditions is statistically significant, we can safely

attribute any male bias to the pronoun itself. Even if the neutral control condition is biased towards men

due to a more general male bias (Hamilton, 1991; Irmen, 2007), we would be able to isolate the male

bias of the pronoun by comparing the control to the experimental conditions in the analysis.

Furthermore, by adding control conditions for the stereotypically male and female experimental

conditions as well, we would be able to tease apart the possible effect of the pronoun from any (added)

effect of the stereotypes, shedding further light on how these two different types of gender cues

interact.”

*Since Experiment 1 is in fact a replication – with some modifications – of Redl et al. (2018), which*

*yielded different results, I could not help wondering about the power of the study. This is especially*

*relevant given a large number of conditions (6 experimental + 6 control). Brysbaert & Stevens (2018),*

*for instance, recommend that a properly powered experiment with repeated measures has at least 1600*

*observations per condition. While it is not typical for eye-tracking studies to follow this*

*recommendation, I would like to know what the decision about the number of participants/stimuli was*

*based on.*

As mentioned above, we determined these six experimental and six control conditions to be necessary

in order to unambiguously identify a male bias introduced by *zijn* ‘his’, particularly because Redl et al.

(2018) did not find that very male bias. As described above, we tried to find as many male participants

as possible and that number also partly determined the total number of participants; while our chosen

type of analysis can deal with unequal numbers of observation very well, one still wants to avoid a large

imbalance. We further included as many items in the experiments as we thought was feasible for

participants while still staying attentive and alert. In summary, we tried to maximize the number of

observations, which in our case were 720 per condition. Also note that we analyzed only four conditions

(the two neutral experimental and two neutral control conditions) as an initial step, since our priority

was to determine whether *zijn* ‘his’ caused a male bias at all. We only extended the analysis if this initial

mode yielded significant results. Since the male bias only showed for male participants, the analysis

was extended to 6 conditions and never to the full 12 conditions. We further made sure to conduct a

stringent analysis by correcting for multiple comparisons to avoid a Type I error (which is often not

done in eye-tracking studies).

*The authors mention that data were log-transformed. However, they do not mention what motivated*

*this particular transformation. What procedure was used to determine which transformation is most*

*beneficial for their data to render it more normal (e.g. Box-Cox)?*

We inspected histograms of the data, which showed a clear right skew as is usual for eye-tracking data.

We then performed a log transformation, plotted a histogram of the transformed data to check whether

the data are more normally distributed (which they were). Of course, the data per se do not need to be

normally distributed, but the residuals do. We thus also visually inspected the residuals of each model

by means of Q-Q plots. We added this information to the paper in the Analysis section under example

4.

*The authors make an important point in the Discussion (lines 716-717) stating that “a sensitive method*

*is needed… for the male bias… to show”. Although they do not elaborate on this any further, it raises*

*a potential issue for the methodological approaches in the study of gender biases in general. In fact,*

*some gender biases have previously been overlooked due to their subtle nature (e.g., Esaulova, Reali,*

*& von Stockhausen, 2017). Importantly, simply because some research tools are not sensitive enough*

*to capture these biases (e.g., sentence evaluation in Experiment 2), does not mean that they are not*

*there. This is why I am not convinced by the authors concluding that “the generic reading is often*

*accessed, too”. This issue should certainly be extended in the discussion.*

We extensively adapted the General discussion based on this valuable comment. We elaborated on why

we think the effect only surfaced using a highly sensitive method such as eye-tracking. We are also

more careful in concluding that the generic reading can be readily available. However, we also

elaborated on why this could be possible in Dutch.

*Minor comments:*

*- lines 114-115: please make explicit why introducing the connective that indicated membership status*

*was important (to establish a reference?).*

We elaborated on this under the heading “Experiment 1: Eye-tracking during reading” when discussing

the changes made to the design by Redl et al (2018):

“This membership had not been made explicit in the stimuli of Redl et al. It is therefore theoretically

possible that the individual in the second sentence was not interpreted as being part of the group referred

to by *zijn* ‘his’ in their experiment. The male bias of the pronoun would then not arise when referring

to a female individual in the second sentence.”

*- line 139: please explain how exactly control conditions allowed to more clearly identify a possible*

*effect of the masculine pronoun.*

We also elaborated on this right underneath example (3) (see also above).

*- line 179: in the* Changed accordingly.

*- line 191: “had a mean rating of 3” - please specify according to which scale.* Changed accordingly.

*- line 208: explain the role of control items (to provide a baseline?)*

We hope to have made this clear with the explanation we provided underneath the example in (3).

*- line 228: participants’ number is stated as 121 here but 120 in the abstract, please be consistent.*

The number of participants stated in the abstract is the number of participants whose data could actually

be analyzed (i.e., after removing participants who did not meet exclusion criteria). We moved the

numbers regarding participant exclusion to the participant section to make this clearer.

*- line 278: please explain why a male bias would be most clearly seen in neutral stereotype contexts*

*(and not in female)*

We changed this to:”… we first only analyzed the neutral stereotype contexts, as a male bias could be

most easily isolated and seen in these contexts if present at all.” Here again, we hope that this is also

made clearer by the explanation added under example (3). But we agree that the formulation “most

clearly seen” was slightly misleading, as a male bias surfacing in female stereotype contexts would be

indicative of a strong effect of the pronoun.

*- line 304: add “(FDR)” here after “false discovery rate” before using the abbreviation later on*

Changed accordingly.

- *lines 343-345: “Notably,…” – please check grammar here*

We hope this is solved by reversing the order: ”Notably, we should see higher reading times for the

word *women* across all stereotype contexts in the *zijn* ‘his’ conditions if the male bias had persisted

throughout all stereotype contexts.”

*- line 349: not a lead* Changed accordingly.

*- lines 497-501: the sentence starting with “Even if…” is too long, please rephrase*

Rephrased into two separate sentences.

*- line 594: add “order” after “randomized”* Changed accordingly.

*- line 608 & 609: replace “second sentence” with “second clause”* Changed accordingly.

**References**

Backer, M. De, & Cuypere, L. De. (2012). The interpretation of masculine personal nouns in German

and Dutch: A comparative experimental study. *Language Sciences*, *34*(3), 253–268.

https://doi.org/10.1016/j.langsci.2011.10.001

Benjamini, Y., & Hochberg, Y. (1995). Controlling the false discovery rate: A practical and powerful

approach to multiple testing. *Journal of the Royal Statistical Society: Series B*, *57*(1), 289–300.

Gabriel, U., & Gygax, P. (2008). Can societal language amendments change gender representation?

The case of Norway. *Scandinavian Journal of Psychology*, *49*(5), 451–457.

https://doi.org/10.1111/j.1467-9450.2008.00650.x

Garnham, A., Gabriel, U., Sarrasin, O., Gygax, P., & Oakhill, J. (2012). Gender representation in

different languages and grammatical marking on pronouns: When beauticians, musicians, and

mechanics remain men. *Discourse Processes*, *49*(6), 481–500.

https://doi.org/10.1080/0163853X.2012.688184

Gygax, P., Gabriel, U., Sarrasin, O., Oakhill, J., & Garnham, A. (2008). Generically intended, but

specifically interpreted: When beauticians, musicians, and mechanics are all men. *Language and*

*Cognitive Processes*, *23*(3), 464–485. https://doi.org/10.1080/01690960701702035

Gygax, P. M., Elmiger, D., Zufferey, S., Garnham, A., Sczesny, S., von Stockhausen, L., Braun, F., &

Oakhill, J. (2019). A language index of grammatical gender dimensions to study the impact of

grammatical gender on the way we perceive women and men. *Frontiers in Psychology*, *10:1604*.

https://doi.org/10.3389/fpsyg.2019.01604

Hamilton, M. C. (1991). Masculine bias in the attribution of personhood: People = Male, Male = People.

*Psychology of Women Quarterly*, *15*(3), 393–402. https://doi.org/10.1111/j.1471-

6402.1991.tb00415.x

Irmen, L. (2007). What’s in a (role) name? Formal and conceptual aspects of comprehending personal

nouns. *Journal of Psycholinguistic Research*, *36*(6), 431–456. https://doi.org/10.1007/s10936-

007-9053-z

Irmen, L., & Schumann, E. (2011). Processing grammatical gender of role nouns: Further evidence

from eye movements. *Journal of Cognitive Psychology*, *23*(8), 998–1014.

https://doi.org/10.1080/20445911.2011.596824

Kliegl, R., & Laubrock, J. (2017). Eye-movement tracking during reading. In A. M. B. De Groot & P.

Hagoort (Eds.), *Research methods in psycholinguistics and the neurobiology of language: A*

*practical guide* (pp. 68–88). Wiley-Blackwell.

Redl, T., Eerland, A., & Sanders, T. J. M. (2018). The processing of the Dutch masculine generic zijn

‘his’ across stereotype contexts: An eye-tracking study. *PLoS ONE*, *13*(10), e0205903.

https://doi.org/10.1371/journal.pone.0205903

Vervecken, D., & Hannover, B. (2015). Yes I can! Effects of gender fair job descriptions on children’s

perceptions of job status, job difficulty, and vocational self-efficacy. *Social Psychology*, *46*(2),

76–92. https://doi.org/10.1027/1864-9335/a000229
