# Supplementary material for: The male bias of a generically-intended masculine pronoun: Evidence from eye-tracking and sentence evaluation
Source: PLoS One. 2021 Apr 1;16(4):e0249309. doi: 10.1371/journal.pone.0249309 (PMC8016286; doi:10.1371/journal.pone.0249309)
Supplement: Attachment — Submitted filename: response-to-reviewers.docx [file pone.0249309.s010.docx]

We repeated all comments below in gray italics font and responded to them individually. Our responses are in black regular font.

***Editor’s comments:***

*... Reviewer #1 still has some concerns…Their main concern is your use of "everyone" in the context sentences. While you have explained at some length why you did not use role nouns - and I think your reasons for this are perfectly valid - the reviewer remains concerned about the inclusive nature of "everyone". I agree with the reviewer that because of this inclusive reading, your context sentences should not be presented as neutral context sentences, and that you should change the framing of your study accordingly.*

We have adapted the manuscript accordingly. In short, we have clarified that when we refer to *neutral contexts* we always mean *stereotypically neutral contexts* as opposed to stereotypically female and male contexts*.* This distinction is based on our extensive pre-test. Mentions of *no gender context being provided* were removed, so as to not give the impression that we were talking about anything but the stereotype contexts. In addition, we address the inclusive nature of *everyone* throughout the paper and extensively elaborated on this in the general discussion, too.

***Reviewer 1’s comments***

*There is still, to me, an issue about the use of “Everyone” in the priming sentence…It’s not so much that I think the materials are not adequate, or that they should replace "everyone" with role nouns, but I think that the way that some of the arguments and some of the interpretation are framed need to be changed.*

*In a nutshell, I think that the authors should carefully think about the difference between a “neutral context” (i.e., no gender) and an “inclusive context” (i.e., both gender, or all genders for that matter). My impression is that the authors think that they tested the first context, whereas I would argue that they tested the second. As such, I think that the paper is still very interesting, as it means that when the context is explicitly made inclusive (by using “everyone”), readers can consider the masculine pronoun as generic (actually, not all readers, as men still struggle). Whether the masculine form can still be considered generic in a neutral context (i.e., when absolutely no gender context is present), this is still to be tested. Let me explain my argument:*

*The authors say already in the abstract “no other gender information is provided”, but I think that this is somehow wrong.*

We clarified this in the abstract:

“Taken together, the results suggest that the generically-intended masculine pronoun *zijn* ‘his’ can cause a male bias for male participants even when the referents are previously introduced by inclusive and grammatically gender-unmarked *iedereen* ‘everyone’.”

*There IS some gender information in the primes, i.e., “everyone”. So the gender information provided is INCLUSIVE, i.e., all genders (and not “no gender”). This is why most gender inclusive language guides in gender marked languages probably include “everyone” as an inclusive form. Maybe the authors misunderstood me the first time, but I am still a little surprised that the authors would not consider “Everyone” as an explicit gender inclusive prime. This is probably why readers show early male bias effect, that may then be overcome by the gender information provided by everyone. As such, the title of the paper should be: “The male bias introduced by the masculine form when primed as generic”. This is very important. In fact, the term “prime” is important. In the same way, on page 7, line 120, when presenting Redl et al., the authors should say that the possessive pronoun was “…in a generically-intended priming contexts…”. The fact that the sentences presented “everyone” before “his” is priming inclusiveness (and not neutrality).*

Thank you for elaborating on this point. We changed the framing of the study to better reflect that the use of *iedereen* ‘everyone’ might have biased readers towards an inclusive reading. Note, however, that we want to avoid the term *priming* as this is usually used somewhat differently in psycholinguistics. It is most often used in the context of semantic or structural priming, which does not apply to our study. As suggested, we presented *everyone* as an inclusive pronoun and nuanced claims beyond these contexts:

**Abstract:** “….Taken together, the results suggest that the generically-intended masculine pronoun *zijn* ‘his’ can cause a male bias for male participants even when the referents are previously introduced by inclusive and grammatically gender-unmarked *iedereen* ‘everyone’…”

**Introduction**, when discussing Redl et al. (2018): “As explained above, the masculine grammatical gender enjoys a wide-reaching default status in Dutch, which might make the generic reading more readily available in online processing. The generic potential of *zijn* ‘his’ might have been boosted by the fact that the referents were first introduced by gender-unmarked and inclusive *iedereen* ‘everyone’, after which the pronoun *zijn* ‘his’ was introduced and anaphorically linked to *iedereen* ‘everyone’.”

**Introduction,** when discussing the hypotheses: “If, however, we were to replicate the null results found by Redl et al., this would add to the evidence suggesting that the masculine gender of *zijn* ‘his’ does not trigger a gender inference and that the pronoun can in fact function as a true generic, at least when used in combination with the indefinite pronoun *iedereen* ‘everyone’.”

**Experiment 1: Eye-tracking during reading**: “Since *zijn* ‘his’ is often used generically in combination with the indefinite pronoun *iedereen* ‘everyone’ [see Chapter 1 in 1 for numerous corpus examples], the use of such stimuli allows us to test if the intended reading in such frequently occurring contexts is actually achieved. As mentioned above, introducing the referents by means of grammatically gender-unmarked and inclusive *iedereen* ‘everyone’ and therefore using the pronoun *zijn* ‘his’ anaphorically, might facilitate a generic interpretation of the masculine pronoun. Thus, if a male bias is found in the contexts we used, then this strongly suggests that the male bias would also surface in other, less inclusive contexts, for example when reversing the order of *iedereen* ‘everyone’ and *zijn* ‘his’ (i.e., a cataphoric – as opposed to anaphoric – use of z*ijn* ‘his’).”

….

“The combination of *ze* ‘they’ with *allemaal* ‘all, altogether, everybody’ as the antecedent was intended to convey a similarly inclusive meaning as *iedereen* ‘everyone’. The possessive pronoun *hun* ‘their’ in the control condition, however, is not marked for grammatical gender.”

**Experiment 2: Sentence evaluation:** “The results from Experiment 1 suggest that – on average – female language users are capable of processing the masculine possessive pronoun *zijn* ‘his’ as gender-neutral in the contexts we presented…”

**General discussion:** “Conversely, this suggests that the generic reading of the generically-intended masculine pronoun *zijn* ‘his’ may be readily available for women, at least in contexts similar to the ones we tested.”

…

“It is important to note at this point that it was a conscious choice to focus on the possessive pronoun *zijn* ‘his’ – as opposed to, for example, the personal pronoun *hij* ‘he’. We assumed that if we found *zijn* ‘his’ to cause a male bias, particularly in the contexts we used, then such a male bias was very likely to be found for a more salient pronoun such as *hij* ‘he’ as well. This is due to the assumed lower salience of *zijn* ‘his’ as already discussed by Redl et al. [2] and discussed in more detail by Redl et al. [3]. In short, Redl et al. [3] note that a personal pronoun such as *hij* ‘he’ and also the role nouns as they were used in the experiments described above [e.g., 4–7] always function as the subject of a clause. The possessive pronoun *zijn* ‘his’, however, functions as a determiner and is dependent on the head of the noun phrase (e.g., *zijn veters* ‘his shoelaces’). Due to this dependency, the possessive pronoun has been shown to be a less salient cue of the referent’s gender [8]. The male bias is therefore less likely to arise based on a masculine possessive pronoun. The fact that we did find such a male bias for *zijn* ‘his’, albeit only for male participants in stereotypically neutral contexts and when using eye-tracking, suggests that more salient generically-intended masculine words such as *hij* ‘he’ would also lead to a male bias, possibly even a larger one. This is indeed what the results by Redl et al. [3] suggest. They found that generically-intended *hij* ‘he’ led to a male bias for male as well as female participants in similar contexts such as the ones used in this experiment. In addition, this lower salience could also partly explain this smaller male bias effect of *zijn* ‘his’ compared to research on role nouns in German and French [e.g., 4–7].

In a similar vein, the antecedent of *zijn* ‘his’ could have further increased the masculine pronoun’s generic potential. In our stimuli, the referents were introduced by *iedereen* ‘everyone’, which also functioned as the subject of the sentence. The pronoun *zijn* ‘his’ was then anaphorically used to link back to the antecedent. The fact that the group was initially introduced as *everyone*, a grammatically gender-unmarked and inclusive pronoun, might have attenuated the male bias of *zijn* ‘his’. It would be interesting to test this in future research. One could, for example, test the male bias of *zijn* ‘his’ when it is used cataphorically, for example:

1. *Na zijn veters gestrikt te hebben, was iedereen klaar voor het vertrek. Een paar van de vrouwen/men waren zo goed als klaar.*

‘After having tied his shoelaces, everyone was ready to leave. Some of the women/men were almost ready.’

Both *iedereen* ‘everyone’ as well as the female and male continuations could then serve as regions of interest, possibly exposing a male bias of *zijn* ‘his’ that is larger than the one found in our experiment. However, the pronoun *everyone* clearly cannot fully attenuate a gender bias; our male participants experienced it in the eye-tracking experiment after all. Furthermore, gender stereotypes also affected processing, even when the referents were introduced by inclusive *everyone* and the gender stereotype information only being provided later [2]. Moreover, it has been previously suggested that supposedly gender-neutral words such as people or gender-unmarked role nouns can lead to a male bias, too [5,9–11]. Thus, even if *everyone* is grammatically inclusive as it can be used to refer to all genders, it might still carry a male bias due to androcentrism in society [12].

Crucially, generically-intended masculine pronouns often have male-biased antecedents in the form of role nouns, as in the example from the newspaper we provided at the very beginning. A male bias could also be more likely to arise in such contexts (e.g., *Een student moet zijn scriptie op tijd indienen* ‘A student (masc.) has to hand in his thesis on time’). To sum up, the stimuli we used likely made it harder for a male bias to arise due to the use of the possessive pronoun *zijn* ‘his’ as well as the indefinite pronoun *iedereen* ‘everyone’ as its antecedent. Thus, the small male bias we found in the current experiment suggests that the male bias of generically-used pronouns may in fact be stronger in other, less inclusive contexts.”

…

*When you think about it, it’s very much like the second study of Duffy and Keir, in which the authors present some context that enables readers to go beyond stereotypes. So in the present experiment, the authors present a strong inclusive context (and not a neutral context, meaning no gender) that enables readers to go beyond the maleness activated by the grammatical form.*

We agree with the reviewer’s general point about the inclusivity of *everyone.* However, the second experiment by Duffy and Keir substantially differs from our experiment. Duffy and Keir explicitly stated whether the referent was female or male – and that was the context enabling readers to go beyond stereotypes. Reading times were then measured after the referent had already been identified as a woman or a man (analyzed regions are in square brackets):

“The electrician was a cautious woman who carefully secured her ladder to the side of the house before checking the roof….The [electrician] [taught] [herself/himself] [a lot while fixing] the problem”

Thus, the electrician is unambiguously identified as female (or in other stimuli as male). The pronoun *everyone* can be said to bias towards inclusivity, but does not unambiguously identify the referents’ gender like in Duffy and Keir’s experiment.

*I also bet that if you were to actually inverse somehow the order “everyone – his” to “his – everyone”, there would be a slow down on everyone (i.e., going from masculine to inclusive), showing that everyone IS a gender cue (i.e., it sets a gender context). My point is that “Everyone” is a biasing gender information. It’s not a problem per se, and this is why in gendered languages “everyone” is at times presented as a good inclusive option when referring to people.*

This is an interesting idea for a follow-up experiment and we included this in the General discussion (see above).

*Now, a TRUE absence of “other biasing gender information” would be a sentence like (a):
(a) “Sitting on the stairs tying his shoelaces, Maaike (f)/Stefan (m) was also getting ready…”*

*Essentially, I have the feeling that the authors mistake the activation of an “inclusive gender context” (or inclusive context) with a “no gender context”. If sentence (a) is not possible in Dutch, this means that the masculine pronoun can only be considered as generic IF (and only if) a strong gender inclusive prime such as “everyone” is presented, but not in a no gender context, because in this case, "his" has to be followed by a masculine name…*

Indeed, as we already tried to explain in the previous round of reviews, a sentence such as in (a) is unsuited to test if *zijn* ‘his’ leads to a male bias when used generically. To test whether a generically-intended pronoun leads to a male bias or can successfully be interpreted as generic, one must present participants with a context in which the masculine pronoun is commonly used generically. Masculine pronouns are commonly used generically in combination with role nouns (*De gemiddelde student woont vaak nog bij zijn ouders* ‘The average student often still lives with his parents’) as well as with indefinite pronouns (*iedereen, iemand* ‘everyone, someone’). Thus, because these are contexts in which the pronoun is actually used generically, one can test whether this intended interpretation is actually achieved. We also emphasized this in our manuscript, as already mentioned above:

“Since *zijn* ‘his’ is often used generically in combination with the indefinite pronoun *iedereen* ‘everyone’ [see Chapter 1 in 1 for numerous corpus examples], the use of such stimuli allows us to test if the intended reading in such frequently occurring contexts is actually achieved.”

The coreferential reading between the pronoun and the female subject in a sentences such as (a) simply cannot arise. The only possible reading for a sentence such as (a) with a female subject, is that Maaike (F) was tying a man’s shoelaces.

While the impossibility of a generic reading of a masculine pronoun in sentences such as (a) is interesting in itself, it would be outside the scope of the present paper to further elaborate on that. We hope we have sufficiently addressed this issue in the revised version of the paper.

*General comment:*

*Before giving some rather minor points, I would like to stress another issue that I have also found in the literature on this topic. I think that we should stop using the terms “masculine generics” in this literature. I know it has been used very often (and by our lab too), but I think (now) that this is fundamentally wrong. Essentially, there is no such thing as a “masculine generic pronoun” (or noun). There is a masculine pronoun (or noun), that’s it. Now, it can be interpreted as generic or as specific, but there is no difference between what would be called a “masculine generic pronoun” or a “masculine specific pronoun”. Both are the same lexical item. I think Hamilton (1988) was wrong to name it this way. It’s highly misleading for readers that do not have grammatical gender in their languages. They will actually think that there are two lexical items (like “han” and “hen” in swedish). There’s only one, the “masculine form”. I hope that the authors see what I mean. In all mentions of "masculine generic form", the authors could simply take out the "generic".*

We have removed all mentions of “masculine generics” from the paper, opting for “generically-intended masculine pronouns/role nouns/words”. The title was changed accordingly.

*Minor comments:*

*1. P4:L50: I find it odd that the authors refer to Norwegian, as to my understanding at least (and according to Gygax et al., 2019 and others), Norwegian is special, as the masculine form was dropped in the 1980’s. If I am not mistaking, this was not the case in Swedish (which adopted a third – neutral - pronoun)*

We are introducing the Dutch grammatical gender system in general here, and not only in reference to nouns referring to humans such as role nouns. The statement that the three-way noun distinction has developed into a two-way system is thus correct. However, we removed Norwegian from the comparison in order to avoid confusion:

“Similar to some Scandinavian languages such as Swedish, the original Indo-European three-way distinction between feminine, masculine and neuter gender has largely developed into a two-way system distinguishing between common gender (comprising formerly masculine and feminine nouns) and neuter gender.”

*2. P5:L56-57: The authors mean ‘female writer’, ‘female teacher’…right? Or maybe I missed something.*

We clarified this:

“Furthermore, even though role nouns formally carry common gender, an unambiguously feminine form can often be derived by attaching a feminine suffix such as *-ster* (e.g., *schrijver* ‘writer’*🡪 schrijfster* ‘female writer’), *-es* (e.g., *leraar* ‘teacher’*🡪 lerares* ‘female teacher’) or *-e* (e.g., *student* ‘student’*🡪 studente* ‘female student’).

*4. P6:L85: I don’t understand the sentence. Did the author mean “…tested the effect of gender stereotype information and the effect of the masculine form on the representation of gender”?*

Changed accordingly:

“With their design, they followed studies on generically-intended masculine role nouns, the majority of which simultaneously tested the effect of gender stereotype information and the effect of the role nouns’ masculine gender on the mental representation of the referent’s gender [e.g., 4,5,7,13–15].”

*5. P6:L86: I would just put “…similar to the grammatical gender – the gender stereotypicality…”*

*Changed accordingly:*

*“*Similar to the grammatical gender of generically-intended masculine words, the gender stereotypicality of role nouns (e.g., *a carpenter is male*) has been shown to be readily used to infer a referent’s gender.”

*6. P6: L91: “…the stereotype OF THE ROLE NOUN, as..” and not of the verb.*

Changed accordingly.

*7. P6: L93: Remove “actual”. Same on line 103.*

Changed accordingly.

*8. P7: L112: The references 34-36 are somehow not really related to Spanish, right?*

The references are indeed not about Spanish, but include additional experiments supporting the conclusion from the Carreiras et al. study. We added the information to the reference to make this clearer:

“This shows that stereotype information is automatically processed as soon as it becomes available, even in the presence of more reliable gender cues. However, once this mismatch is resolved, pronouns mismatching the stereotype (but matching the previously established referent’s gender) do not lead to processing difficulties anymore [for further evidence of the automatic activation of gender stereotypes from English see 16–18].”

*9. P19:L420-422: I would say that this is an empirical question. By saying this, does it mean that the authors did analyse regression path to the first clause? And that the results were the same.*

We changed the sentence to make this clearer:

“All analyzed regions are in the second clause. We chose to focus on the second clause and not to analyze measures of regression into regions in the first clause, because regression path duration on the three chosen regions by definition – as it is provided above – already capture regressions into the first clause.”

By definition, the regression path duration of a certain region contains all first pass fixations on that region, plus all regressions back to earlier regions that were launched from the region in question. We also provide this definition in the paper:

“first run dwell time (i.e., the sum of the duration of all fixations in a region when it is entered for the first time, also known as gaze duration), regression path duration (i.e., first run dwell time with the addition of the duration of fixations back to previous regions out of the analyzed region) and dwell time (i.e., the sum of the duration of all fixations in a region, also known as total fixation duration).”

For example, the regression path duration on the pre-view region *enkele/een paar* ‘some/a few’ thus includes all first pass fixations on that region, plus all fixations on words before that region that were launched after reading *enkele/een paar* ‘some/a few’ for the first time. More specifically, regression path duration on ‘some/a few’ includes regressions back to the words in bold below:

2. **Iedereen was zijn schoenen aan het aandoen, waaronder** [enkele/een paar]_1_ [vrouwen/mannen]_2_ [die al]_3_ bijna klaar waren om de deur uit te gaan, maar een beetje aan het treuzelen waren.
   ‘**Everyone was putting on his shoes, among whom** [some/a few]_1_ [women/men]_2_ [who already]_3_ were almost ready to go out, but were still slacking.’

We hope that this is clearer now.

*Given the rather lack of effects, I think it would be reassuring to know that the results are the same (even by presenting some Supplementary analysis in a different file).*

We included two additional analyses as Supplemental material 8. It is referenced in the paper underneath the paragraph quoted above:

“Exploratory analyses on the number of regressions in the first clause were conducted after the main analysis and can be found in the Supplemental material.”

*10. P19:L429: I do find it rather odd that the analysis was conducted separately across stereotype groups. Did Redl et al. do the same?*

We further elaborated on our decision to conduct a two-step analysis:

“As the primary research question of this experiment was whether the possessive pronoun *zijn* ‘his’ leads to a male bias, we decided to first only analyze the neutral stereotype contexts, as a male bias could be most easily isolated and seen in these contexts if present at all. This choice was made in order to simplify the analysis. Due to the presence of four factors and twelve conditions in total, four-way interactions could possibly arise, but they are notoriously hard to interpret. Note that Redl et al. [2] only had three possible factors and six conditions due to the absence of elaborate control conditions. Our approach is thus different from their statistical analysis. In a first step, participant gender (*female* versus *male*), pronoun (*zijn* ‘his’ versus *hun* ‘their’) and continuation (*vrouwen* ‘women’ versus *mannen* ‘men’) served as fixed effects. Only if a male bias was found (i.e., an interaction between pronoun and continuation*,* such that the reading time increase for female compared to male continuations is larger on experimental than on control items) for either or both participant genders (i.e., for all participants, or only for male participants or – though we hypothesized this to be less likely – only for female participants), did we extend the analysis to stereotypically male and female contexts as well. This was done to investigate if the male bias of the pronoun persists in otherwise gendered contexts. If no male bias was found in the neutral context, we did not extend the analysis to female and male stereotype contexts.”

*And is this why the authors used the Benjamini and Hochberg procedure. This could be a little clearer I guess.*

Note that the approach described above has nothing to do with applying the Benjamini and Hochberg procedure. We elaborated on this in the paper and added a reference showing why such a procedure is crucial:

“We followed Benjamini and Hochberg [19] and applied their false discovery rate (FDR) control in order to correct for conducting multiple tests, as we analyzed three reading time measures in three regions, leading to nine models or tests. The inflated Type I error rate caused by multiple comparisons is traditionally ignored in eye-tracking research, but Von der Malsburg and Angele showed [20] with Monte Carlo simulations that “false positives are increased to unacceptable levels when no corrections are applied” (p.133). We therefore applied the correction by Benjamini and Hochberg in order to reduce the chance of a false positive.”

References:

[1]	Redl T. Masculine generic pronouns: Investigating the processing of an unintended gender cue (MPI Series in Psycholinguistics Nr 162) [Doctoral thesis]. Radboud University; 2021.

[2]	Redl T, Eerland A, Sanders TJM. The processing of the Dutch masculine generic zijn ‘his’ across stereotype contexts: An eye-tracking study. PLoS One. 2018;13(10):e0205903. https://doi.org/10.1371/journal.pone.0205903

[3]	Redl T, Frank S, de Swart P, de Hoop H. The male bias of a generically-intended personal pronoun in language processing. PsyArXiv. 2020; https://doi.org/10.31234/osf.io/3hc69

[4]	Gygax P, Gabriel U, Sarrasin O, Oakhill J, Garnham A. Generically intended, but specifically interpreted: When beauticians, musicians, and mechanics are all men. Lang Cogn Process. 2008;23(3):464–85. https://doi.org/10.1080/01690960701702035

[5]	Irmen L. What’s in a (role) name? Formal and conceptual aspects of comprehending personal nouns. J Psycholinguist Res. 2007;36(6):431–56. https://doi.org/10.1007/s10936-007-9053-z

[6]	Misersky J, Majid A, Snijders TM. Grammatical gender in German influences how role-nouns are interpreted: Evidence from ERPs. Discourse Process. 2019;56(8):643–54. https://doi.org/10.1080/0163853X.2018.1541382

[7]	Garnham A, Gabriel U, Sarrasin O, Gygax P, Oakhill J. Gender representation in different languages and grammatical marking on pronouns: When beauticians, musicians, and mechanics remain men. Discourse Process. 2012;49(6):481–500. https://doi.org/10.1080/0163853X.2012.688184

[8]	Slevc LR, Wardlow Lane L, Ferreira VS. Pronoun production: Word or world knowledge? MIT Work Pap Linguist. 2007;53:191–203.

[9]	Hamilton MC. Masculine bias in the attribution of personhood: People = Male, Male = People. Psychol Women Q. 1991;15(3):393–402. https://doi.org/10.1111/j.1471-6402.1991.tb00415.x

[10]	Heise E. Sind Frauen mitgemeint? Eine empirische Untersuchung zum Verständnis des generischen Maskulinums und seiner Alternativen. Spr Kognition. 2000;19:3–13. https://doi.org/10.1024//0253-4533.19.12.3

[11]	Silveira J. Generic masculine words and thinking. Womens Stud Int Q. 1980;3(2–3):165–78. https://doi.org/10.1016/S0148-0685(80)92113-2

[12]	Hegarty P, Buechel C. Androcentric Reporting of Gender Differences in APA Journals: 1965-2004. Rev Gen Psychol. 2006;10(4):377–89. https://doi.org/10.1037/1089-2680.10.4.377

[13]	Irmen L, Roßberg N. Gender markedness of language: The impact of grammatical and nonlinguistic information on the mental representation of person information. J Lang Soc Psychol. 2004;23(3):272–307. https://doi.org/10.1177/0261927X04266810

[14]	Irmen L, Holt D V., Weisbrod M. Effects of role typicality on processing person information in German: Evidence from an ERP study. Brain Res. 2010;1353:133–44. https://doi.org/10.1016/j.brainres.2010.07.018

[15]	Gabriel U, Gygax P. Can societal language amendments change gender representation? The case of Norway. Scand J Psychol. 2008;49(5):451–7. https://doi.org/10.1111/j.1467-9450.2008.00650.x

[16]	Garnham A, Oakhill J, Reynolds D. Are inferences from stereotyped role names to characters’ gender made elaboratively? Mem Cognit. 2002;30(3):439–46. https://doi.org/10.3758/BF03194944

[17]	Oakhill J, Garnham A, Reynolds DJ. Immediate activation of stereotypical gender information. Mem Cognit. 2005;33(6):972–83. https://doi.org/10.3758/BF03193206

[18]	Osterhout L, Bersick M, McLaughlin J. Brain potentials reflect violations of gender stereotypes. Mem Cognit. 1997;25(3):273–85. https://doi.org/10.3758/BF03211283

[19]	Benjamini Y, Hochberg Y. Controlling the false discovery rate: A practical and powerful approach to multiple testing. J R Stat Soc Ser B. 1995;57(1):289–300.

[20]	von der Malsburg T, Angele B. False positives and other statistical errors in standard analyses of eye movements in reading. J Mem Lang. 2017;94:119–33. https://doi.org/10.1016/j.jml.2016.10.003
